# Small extracellular vesicles derived from interferon-γ pre-conditioned mesenchymal stromal cells effectively treat liver fibrosis

**DOI:** 10.1038/s41536-021-00132-4

**Published:** 2021-03-30

**Authors:** Suguru Takeuchi, Atsunori Tsuchiya, Takahiro Iwasawa, Shunsuke Nojiri, Takayuki Watanabe, Masahiro Ogawa, Tomoaki Yoshida, Katsunori Fujiki, Yuta Koui, Taketomo Kido, Yusuke Yoshioka, Mayu Fujita, Junichi Kikuta, Tohru Itoh, Masaaki Takamura, Katsuhiko Shirahige, Masaru Ishii, Takahiro Ochiya, Atsushi Miyajima, Shuji Terai

**Affiliations:** 1 Division of Gastroenterology and Hepatology, Graduate School of Medical and Dental Sciences, Niigata University, 1-757 Asahimachi-dori, Chuo-ku, Niigata 951-8510 Japan; 2 Laboratory of Genome Structure and Function, Institute for Quantitative Biosciences, The University of Tokyo, 1-1-1, Yayoi-Cho, Bunkyo-ku, Tokyo 113-0032 Japan; 3 Laboratory of Stem Cell Therapy, Institute for Quantitative Biosciences, The University of Tokyo, Tokyo, 113-0032 Japan; 4 Department of Molecular and Cellular Medicine, Institute of Medical Science, Tokyo Medical University, 6-7-1, Nishi-Shinjuku, Shinjuku-ku, Tokyo 160-0023 Japan; 5 Department of Immunology and Cell Biology, Graduate School of Medicine and Frontier Biosciences, Osaka University, 2-2 Yamada-oka, Suita, Osaka 565-0871 Japan

**Keywords:** Mesenchymal stem cells, Mesenchymal stem cells

## Abstract

Mesenchymal stromal cells (MSCs) are used for ameliorating liver fibrosis and aiding liver regeneration after cirrhosis; Here, we analyzed the therapeutic potential of small extracellular vesicles (sEVs) derived from interferon-γ (IFN-γ) pre-conditioned MSCs (γ-sEVs). γ-sEVs effectively induced anti-inflammatory macrophages with high motility and phagocytic abilities in vitro, while not preventing hepatic stellate cell (HSC; the major source of collagen fiber) activation in vitro. The proteome analysis of MSC-derived sEVs revealed anti-inflammatory macrophage inducible proteins (e.g., annexin-A1, lactotransferrin, and aminopeptidase N) upon IFN-γ stimulation. Furthermore, by enabling CX_3_CR1+ macrophage accumulation in the damaged area, γ-sEVs ameliorated inflammation and fibrosis in the cirrhosis mouse model more effectively than sEVs. Single cell RNA-Seq analysis revealed diverse effects, such as induction of anti-inflammatory macrophages and regulatory T cells, in the cirrhotic liver after γ-sEV administration. Overall, IFN-γ pre-conditioning altered sEVs resulted in efficient tissue repair indicating a new therapeutic strategy.

## Introduction

The liver is a highly regenerative organ; however, chronic liver injury caused by hepatitis virus infection, alcoholism, and non-alcoholic steatohepatitis (NASH) results in hepatocyte loss, scar deposition, and ultimately cirrhosis^1^. It is well known that fibrosis can regress spontaneously after therapies such as treatment for hepatitis B and C infections; however, livers with advanced cirrhosis often lose this ability^2,3^. Although many researchers are developing targeted therapies for ameliorating fibrosis and aiding regeneration, clinically advanced cirrhosis is an end-stage disease that can effectively be treated only by liver transplantation at present^4^.

Cell therapy is an option for inducing liver fibrosis regression. Currently, mesenchymal stromal cells (MSCs) and macrophages have attracted the attention of the field and clinical studies are underway^5–12^. MSCs can be obtained not only from the bone marrow, but also from medical waste, including umbilical cord tissue, adipose tissue, and dental pulp; they also have an advantage as they can be cultured relatively easily. MSCs have been characterized as medical signaling cells or conducting cells that act indirectly by producing cytokines, chemokines, growth factors, and exosomes, rather than acting directly by replacing the damaged tissues^5,6^. They can exert anti-inflammatory, anti-fibrotic, and anti-oxidative effects through these humoral factors. In addition, MSCs possess low immunogenicity, which has facilitated their use for both autologous and allogeneic transplantation in more than 900 clinical trials in multiple fields, including the treatment of liver diseases^7^.

Macrophages are the regulators of liver fibrosis and its resolution^10,11^. Although macrophages are heterogeneous in phenotype and function, they can differentiate into two polarities, either pro- or anti-inflammatory^13^. Forbes et al. focused on the ability of macrophages to home in damaged areas, express matrix metalloproteinases (MMPs), and recruit host cells^11^. They have developed a system for isolating circulating CD14+ monocytes from patients with cirrhosis and can differentiate these cells into macrophages using a good manufacturing practice (GMP)-compliant process; indeed, they were the first to initiate human phase I clinical trials for patients with liver cirrhosis^12^.

Recently, our group reported that relative to MSC and macrophage monotherapy, simultaneous treatment with MSCs and bone marrow-derived macrophages synergistically ameliorated liver fibrosis, suggesting that these cell types interact in the body. Most MSCs did not migrate into the liver, but instead migrated to and were trapped in the lung. In contrast, many macrophages homed to damaged areas of the liver, some near collagen fibers or phagocytosing hepatocyte debris^9^. From these observations, we speculated that some cargo contained in MSCs may have influenced the macrophages, and that small extracellular vesicles (sEVs), which are approximately 100 nm-wide EVs formed by the endosomal system and secreted by the MSCs, may play important roles in MSC-macrophage communication. The lipid bilayer-enclosed sEVs are extremely stable and harbor molecules including proteins, RNAs, lipids, and other metabolites^14^. In addition, sEVs from MSCs possess hypoimmunogenic properties, allowing human MSC-derived sEVs (MSC-sEVs) to be used in multiple animal disease models^15,16^.

In this study, we aimed to confirm that MSC-derived sEVs are important for MSC-macrophage communication and investigated appropriate pre-conditioned MSC methods that could produce sEVs with the ability to induce the anti-inflammatory macrophages with high tissue repairability and high therapeutic potential for treating liver fibrosis in a mouse model. Our study represents initial steps in the development of a future therapy.

## Results

### IFN-γ pre-conditioned MSC-derived sEVs effectively induce anti-inflammatory macrophage responses in vitro

**Fig. 1:**
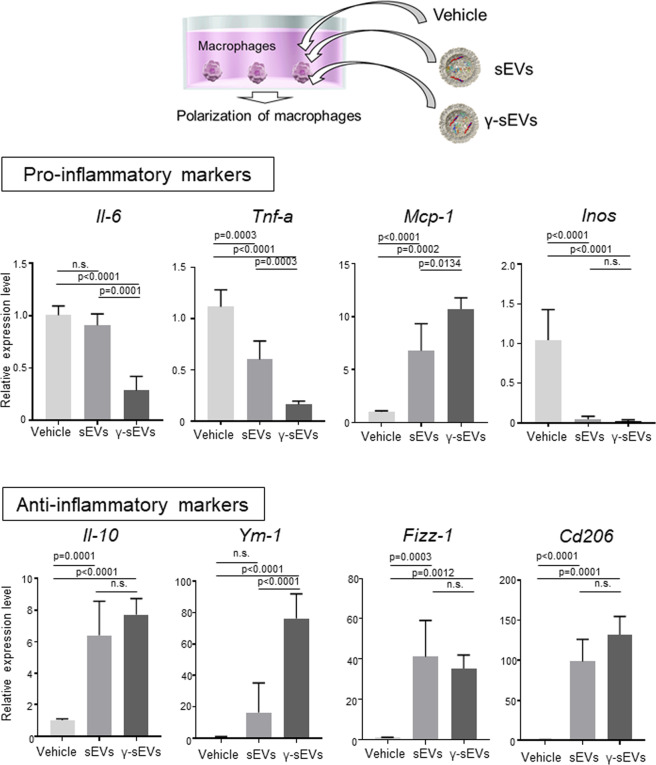
Analysis of changes in macrophage polarity produced by AD-MSC-sEVs (sEVs) or AD-MSC-γ-sEVs (γ-sEVs). Forty-eight hours after the addition of vehicle (PBS), sEVs, and γ-sEVs, macrophages were harvested and the mRNA expression levels of genes encoding pro- (*Il-6, Tnf-a, Mcp-1, Inos*) and anti- (*Il-10, Ym-1, Fizz-1, Cd206*) inflammatory factors were evaluated using real-time PCR. Data are presented as means ± SD; *n* = 5 per experiment.

We analyzed whether sEVs from human adipose tissue-derived MSCs (AD-MSC-sEVs) can affect the polarity (pro- or anti-inflammatory) of bone marrow-derived macrophages (BMMs). As MSCs are known to induce a robust anti-inflammatory response when host inflammation is high, and IFN-γ are often reported to change the character of MSCs under inflammatory conditions, we attempted to harvest sEVs following pre-conditioning with IFN-γ^17^. First, we observed the effect of sEVs on the polarity of macrophages by adding AD-MSC-sEVs or PBS (vehicle control) to BMMs. AD-MSC-sEVs significantly decreased the expression of pro-inflammatory (*Tnf-a* and *Inos*) macrophage factors and increased the expression of both pro-inflammatory (*Mcp-1*) and anti-inflammatory (*Il-10, Fizz-1, Cd206*) macrophage factors. Next, we analyzed the effect of sEVs harvested from IFN-γ pre-conditioned MSCs. The polarity effectively shifted toward anti-inflammatory when IFN-γ pre-conditioned human AD-MSC-derived sEVs (AD-MSC-γ-sEVs) were added to BMMs. The levels of pro-inflammatory macrophage factors (*Il-6, Tnf-a, Inos*) decreased significantly, whereas those of anti-inflammatory macrophage factors (*Il-10, Ym-1, Fizz-1, Cd206*) increased significantly. The levels of the anti-inflammatory macrophage factors Ym-1 in cells treated with AD-MSC-γ-sEVs were significantly higher than those in cells treated with AD-MSC-sEVs (p = 0.0001). In contrast, the levels of the pro-inflammatory macrophage factor *Il-6* in cells treated with AD-MSC-γ-sEVs were significantly lower than those in cells treated with AD-MSC-sEVs (p = 0.0001). In macrophages treated with AD-MSC-γ-sEVs, the proteins levels of TNF-α and MCP-1 decreased significantly compared to those treated with AD-MSC-sEVs (TNF-α; *p* = 0.0456, MCP-1; *p* = 0.0156; Supplementary Fig. 2), indicating that AD-MSC-γ-sEVs can induce the production of anti-inflammatory macrophages more effectively than AD-MSC-sEVs (Fig. 1). Furthermore, macrophages treated with AD-MSC-γ-sEVs had significantly lower levels of nitrites than those treated with AD-MSC-sEVs (*p* = 0.0017, Supplementary Fig. 2). Collectively, these results revealed that AD-MSC-γ-sEVs effectively induce anti-inflammatory macrophage responses in vitro.

### MSC-derived sEVs enhance motility and phagocytic ability

**Fig. 2:**
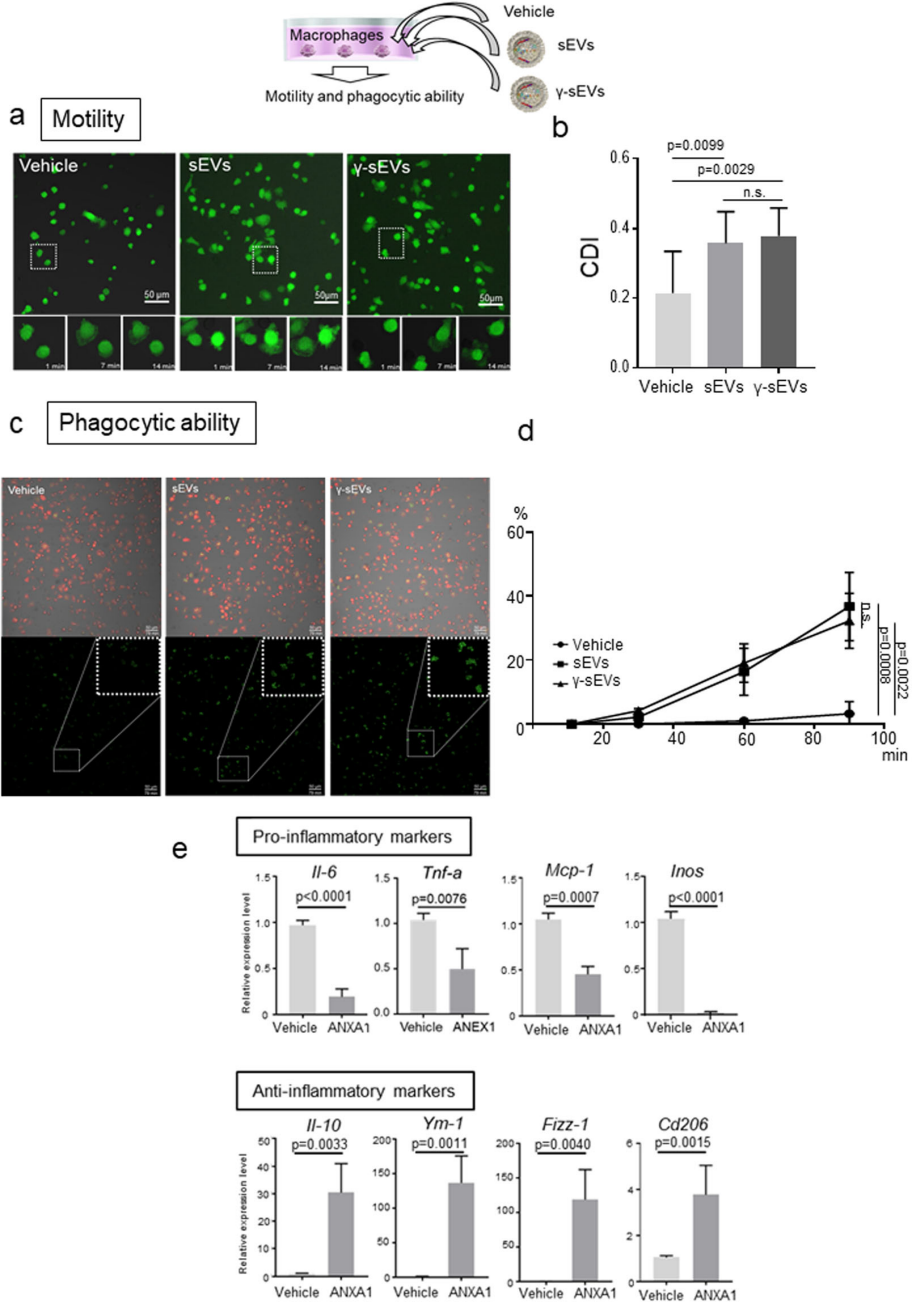
Macrophage motility and phagocytic ability after addition of AD-MSC-sEVs (sEVs) or AD-MSC-γ-sEVs (γ-sEVs). **a** Motility of GFP+ macrophages after addition of vehicle, sEVs, and γ-sEVs (Supplementary Movie 1; vehicle, sEVs and γ-sEVs from left to right). **b** Quantification of morphological changes in macrophages. Data are presented as means ± SD; ten screens were counted in each experiment. CDI, cell deformation index. **c** Phagocytic ability of DsRed+ macrophages after phagocytosis of pHrodo™ Green Zymosan Bioparticles™ conjugate (Supplementary Movie 2; vehicle, sEVs, and γ-sEVs from left to right). **d** Frequencies of green fluorescent macrophages observed using time-lapse imaging. Data are presented as means ± SD; ten screens were counted in each experiment 90 min after the addition of sEVs or γ-sEVs. Scale bar = 50 μm. **e** Change in macrophage polarity after treatment with recombinant human annexin-A1 (ANXA1).

Next, we assessed the effect of AD-MSC-sEVs and AD-MSC-γ-sEVs on macrophage motility and phagocytic activity. For motility, BMMs harvested from GFP-transgenic mice were seeded in culture dishes with PBS (vehicle control), AD-MSC-sEVs, or AD-MSC-γ-sEVs, and serial photographs were captured to evaluate their motility 4 h later. While most of the BMMs incubated with the vehicle did not move, macrophages incubated with AD-MSC-sEVs and AD-MSC-γ-sEVs moved vigorously (Fig. 2a, b, Supplementary Movie 1). We further assessed phagocytic activity by adding pHrodo^TM^ Green Zymosan Bioparticles^TM^, which generate green fluorescence after phagocytosis. While most of the BMMs with the vehicle did not fluoresce, BMMs incubated with AD-MSC-sEVs (*p* = 0.0008 compared to the vehicle) and AD-MSC-γ-sEVs (*p* = 0.0022 compared to vehicle) significantly phagocytosed the particles (Fig. 2c, d and Supplementary Movie 2). These results suggested that both AD-MSC-sEVs and AD-MSC-γ-sEVs promote motility and phagocytic activity.

### MSC-derived sEVs do not prevent the direct activation of hepatic stellate cells in vitro

Activated hepatic stellate cells (HSCs) are the major source of collagen fiber during fibrogenesis. To determine whether AD-MSC-sEVs and/or AD-MSC-γ-sEVs directly inhibited the activation of HSCs, mouse HSCs were harvested and seeded into collagen-coated dishes, which are known to activate HSCs. AD-MSC-sEVs and AD-MSC-γ-sEVs were added on day 1 after seeding. *Acta2, Col1a1*, and *Col3a1* mRNA levels, which increase after HSC activation, were assessed using real-time PCR on day 4 (Supplementary Fig. 3A). The mRNA levels of *Acta2, Col1a1*, and *Col3a1* did not change significantly after addition of AD-MSC-sEVs and AD-MSC-γ-sEVs nor did the frequency of α-smooth muscle actin (SMA) positive cells, suggesting that they did not prevent the direct activation of HSCs (Supplementary Figs. 3B, 4A and 4B). These results suggested that AD-MSC-sEVs and AD-MSC-γ-sEVs do not directly inhibit HSC activation.

### IFN-γ pre-conditioned MSC-derived sEVs contain anti-inflammatory macrophage inducible proteins

Next, we analyzed proteins and miRNAs present in the sEVs. AD-MSC-sEVs and AD-MSC-γ-sEVs contained 46 and 70 proteins, respectively, of which the levels of 53 proteins either increased or appeared after IFN-γ stimulation (Supplementary Table 1). We screened for proteins related to macrophage polarization and selected five proteins: annexin-A1, lactotransferrin, galectin-3-binding protein, lactadherin, and aminopeptidase N. We added each of these recombinant proteins into the BMM culture and observed changes in macrophage polarity using real-time PCR. All five proteins affected macrophage polarization; however, annexin-A1, lactotransferrin, and aminopeptidase N strongly increased the anti-inflammatory macrophage count by decreasing pro-inflammatory factors (*Il-6*, *Tnf-a*, and *Inos*) and increasing anti-inflammatory factors (*Il-10, Ym-1, Fizz-1*, and *Cd206*) (Fig. 2e, Supplementary Figs. 5 and 6).

To analyze the difference in expression of miRNAs between AD-MSC-sEVs and in AD-MSC-γ-sEVs, an MA-plot (a method of visualizing the difference between two samples by transforming the data onto M (log ratio) and A (mean average) scales) was generated. The 10 miRNAs with the highest absolute amount were *MIRLET7B, MIRLET7I, MIR21, MIR199A2, MIR125B1, MIR125B2, MIR199B, MIR199A1, MIR221*, and *MIR16-2*. The top 10 miRNAs that were upregulated after IFN-γ stimulation were *MIR185, MIR1291, MIR339, MIR664-A, MIR377, MIR1248, MIR1306, MIR145, MIR15A*, and *MIR589*. While the roles of these miRNAs have not been confirmed, we observed that their levels did not change significantly after IFN-γ stimulation, and that the miRNAs in AD-MSC-sEVs that significantly increased after IFN-γ stimulation were originally poorly expressed (Supplementary Fig. 7). Overall, the contents of these EVs suggest that the proteins induced by IFN-γ participate in communication between MSCs and macrophages.

### IFN-γ pre-conditioned MSC-derived sEVs effectively improve inflammation and fibrosis in a mouse model of cirrhosis

**Fig. 3:**
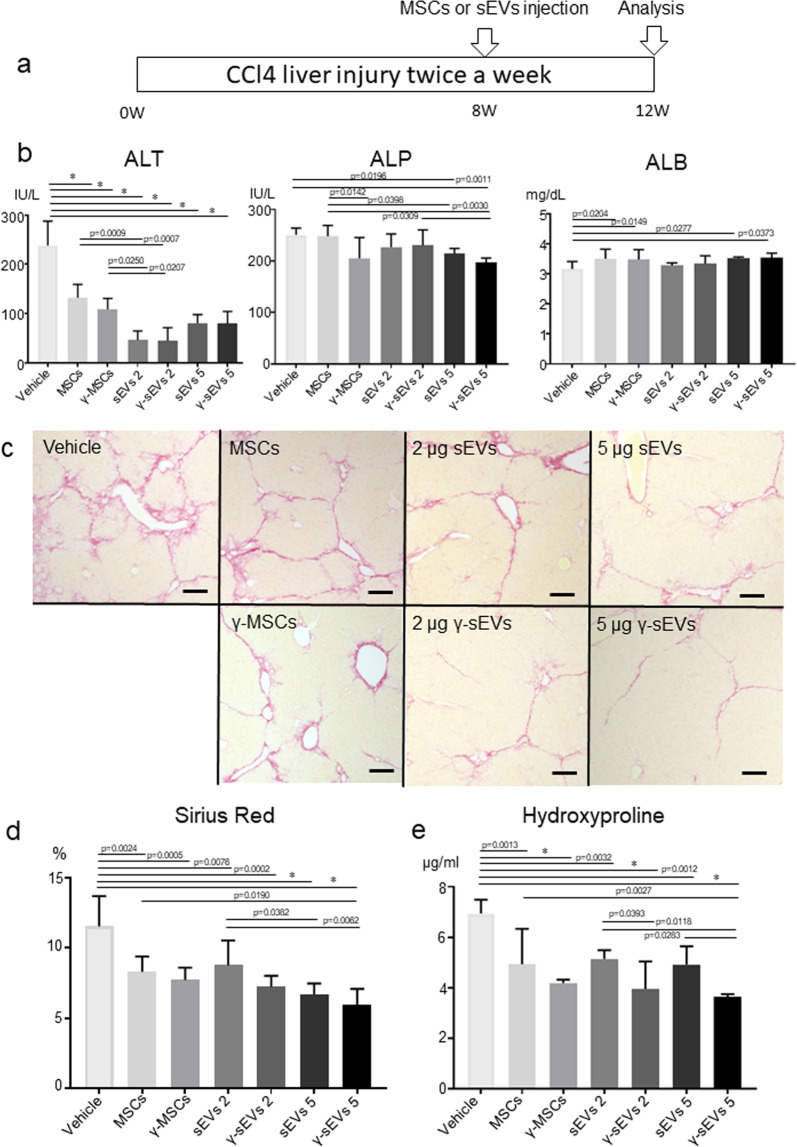
Therapeutic effect of AD-MSC (MSC), γ-AD-MSC (γ-MSC), AD-MSC-sEVs (sEVs), and AD-MSC-γ-sEVs (γ-sEVs). **a** Schematic showing the experimental design for induction of fibrosis, cell or sEV administration, and analysis. **b** Serum levels of ALT, ALP, and ALB. Data are presented as means ± SD; *n* = 5 per experiment. **c** Representative Sirius Red staining of vehicle, MSC, γ-MSC, sEVs2, γ-sEVs2, sEVs5, and γ-sEVs5 groups. **d** Evaluation of fibrosis from Sirius Red-stained areas and **e** quantitation of hydroxyproline levels. Data are presented as means ± SD; *n* = 5 per experiment. *p < 0.0001.

Next, we analyzed the effects of MSCs and their sEVs in a CCl4-induced murine liver cirrhosis model^18^. CCl4 was injected intraperitoneally for 12 weeks. At 8 weeks, PBS (vehicle), AD-MSC, IFN-γ pre-conditioned AD-MSC (γ-AD-MSC), AD-MSC-sEVs, and AD-MSC-γ-sEVs were injected (Fig. 3a). Serum analysis after 12 weeks revealed that alanine aminotransferase (ALT) levels decreased significantly in all the MSC and sEV groups, suggesting that both MSCs and sEVs exerted an anti-inflammatory effect. Furthermore, 2 μg of both AD-MSC-sEVs and AD-MSC-γ-sEVs produced a stronger anti-inflammatory effect than AD-MSCs and γ-AD-MSCs, respectively. In addition, 5 μg of both AD-MSC-sEVs and AD-MSC-γ-sEVs effectively decreased alkaline phosphatase (ALP) levels, and 5 μg of AD-MSC-γ-sEVs (*p* = 0.0011 compared to the vehicle) was more effective than the same amount of AD-MSC-sEVs (*p* = 0.0196 compared to the vehicle). Relative to the vehicle, AD-MSC, γ-AD-MSC, and 5 μg each of AD-MSC-sEV and AD-MSC-γ-sEV significantly increased albumin (ALB) levels (Fig. 3b). While all the MSC- and sEV-injected groups showed an improvement in fibrosis, Sirius Red staining, quantitation of hydroxyproline levels and the αSMA staining area revealed that MSC and sEV therapy achieved the same anti-fibrotic effects. We did not detect clear dose-dependent effects of sEVs; in particular, the quantitation of hydroxyproline revealed that both 2 μg and 5 μg of AD-MSC-γ-sEVs improved liver fibrosis more than the AD-MSC-sEVs (2 μg, *p* = 0.0393; 5 μg, *p* = 0.0283) (Fig. 3c–e, Supplementary Fig. 8). These data suggested that the therapeutic effects of AD-MSC-γ-sEVs were superior to those of AD-MSC-sEVs and indistinguishable from those of MSC therapy.

### IFN-γ pre-conditioned MSC-derived sEVs induce wide-ranging transcriptional changes

**Fig. 4:**
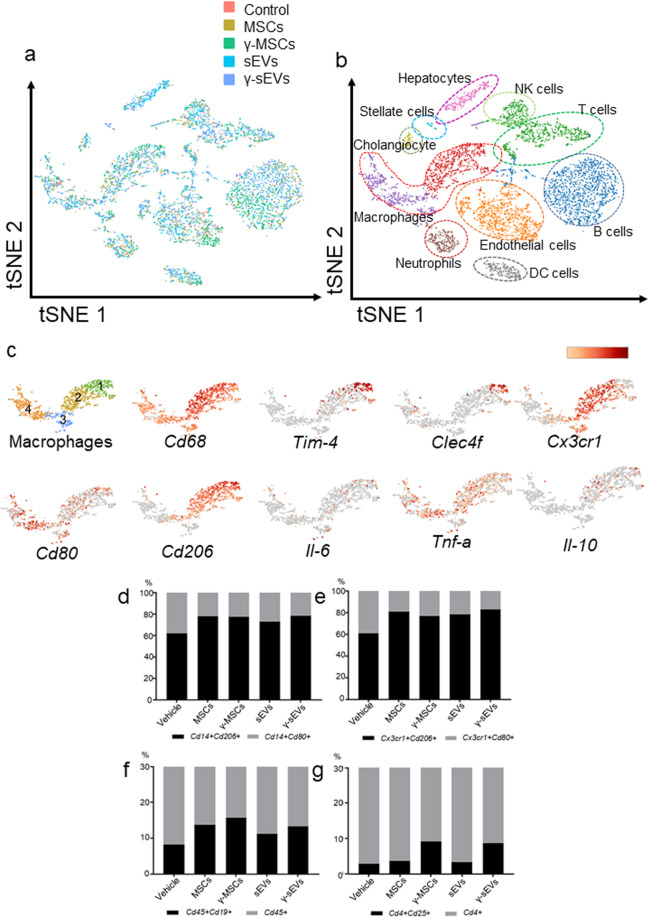
Single-cell transcriptome analysis after injection of AD-MSCs (MSCs), γ-AD-MSCs (γ-MSCs), AD-MSC-sEVs (sEVs), and AD-MSC-γ-sEVs (γ-sEVs). **a** The t-distributed stochastic neighbor embedding (t-SNE) graph plotted by combining data for vehicle (PBS), MSC, γ-MSC, sEVs, and γ-sEVs. Red, brown, green, blue, and purple dots show the vehicle (663 cells), MSC (1215 cells), γ-MSC (1505 cells), sEV (1588 cells), and γ-sEV groups (1591 cells), respectively. **b** Representative cell characters in the clusters shown in a t-SNE plot. **c** Gene expression in selected macrophage populations. These macrophage populations were divided into four groups (1–4). **d–g** Frequency of *Cd14* + *Cd206* + and *Cd14* + *Cd80* + cells (**d**), *Cx3cr1* + *Cd206* + and *Cx3cr1* + *Cd80* + cells (**e**), *Cd45* + *Cd19* + cells in *Cd45* + cells (**f**), and *Cd4* + *Cd25* + cells in *Cd4* + cells (**g**) in the different groups.

Next, we analyzed the effects of MSCs and sEVs on the behavior of hematopoietic cells (mainly macrophages) in the liver, using single-cell RNA-sequencing. Cirrhosis was induced by injecting mice with CCl4 for 8 weeks, followed by injection of PBS (vehicle), AD-MSCs, γ-AD-MSCs, AD-MSC-sEVs (5 μg), and AD-MSC-γ-sEVs (5 μg). Livers were harvested 48 h after the injection. We analyzed 663 cells in the vehicle group, 1215 cells in the AD-MSC group, 1505 cells in the γ-AD-MSC group, 1588 cells in the AD-MSC-sEVs group, and 1591 cells in the AD-MSC-γ-sEV group. We categorized the cells into 10 groups, which included mainly macrophages, neutrophils, B cells, T cells, NK cells, and endothelial cells, as shown in t-distributed stochastic neighbor embedding (t-SNE) and violin plots (Fig. 4a, b, Supplementary Fig. 9). Next, we analyzed the macrophage area in detail. Almost all macrophages were *Cd68+*. These macrophage populations were divided into four groups (macro1-4) as shown Fig. 4c. Kupffer cell markers, *Tim-4* and *Clec4f*, were mainly expressed in macro1, the pro-inflammatory macrophage marker, *Cd80*, was diffusely expressed in all groups but dominantly expressed in macro4, the anti-inflammatory macrophage marker, *Cd206*, was expressed in macro1 and macro2, *Il-6* and *Tnf-α* were diffusely expressed and *Il-10* was mainly expressed in macro1 and macro2, and *Cx3cr1* was expressed in macro1 and macro2 (Fig. 4c). We also checked the distribution of scar-associated macrophages (SAMs; TREM2+CD9+) defined by Ramachandran et al., which were detected in macro1 and macro2 (Supplementary Fig. 10A)^19^. These SAMs expressed *Mmp12*, *13*, *14*, *19*, *27*, and *Timp2* (Supplementary Fig. 11), and increased in abundance after γ-AD-MSCs and AD-MSC-γ-sEVs treatment (Supplementary Fig. 10B). We also confirmed that in SAMs, the *Cd206*+/*Cd80*+ ratio was increased (Supplementary Fig. 10C), while there was a decrease in *Il-1b*+ and *Tnf-a*+ cells in the AD-MSC-γ-sEVs treated groups (Supplementary Fig. 10D and E). We also compared the abundance of *Cd206*+ anti-inflammatory macrophages and *Cd80*+ pro-inflammatory macrophages among the *Cd14*+ and *Cx3cr1*+ cells. In all the MSC and sEV injection groups, *Cd206*+ cells were abundant in both the *Cd14*+ and *Cx3cr1*+ cell fractions (Fig. 4d, e). In addition, in all the MSC and sEV injection groups, the abundance of *Cd19*+ cells increased among *Cd45*+ cells (Fig. 4f). Upon IFN-γ stimulation, the abundance of *Cd4*+ *Cd25*+ cells, which represent regulatory T (Treg) cells in mice, increased among *Cd4*+ cells only in the γ-AD-MSC and AD-MSC-γ-sEV groups, implying that IFN-γ stimulated the MSCs to produce molecules that induce Treg cells (Fig. 4g). These results suggest that single-cell RNA-seq is useful for determining the multidimensional effects of cell therapy and EV therapy. They also indicated that IFN-γ stimulation induced multidirectional effects by not only increasing the abundance of anti-inflammatory macrophages, but also by increasing the abundance of Treg cells. This is supported by other results showing that IFN-γ pre-conditioning can promote tissue repair and reduce fibrosis.

### MSC-derived sEVs cause accumulation of CX_3_CR1+ macrophages in damaged areas

**Fig. 5:**
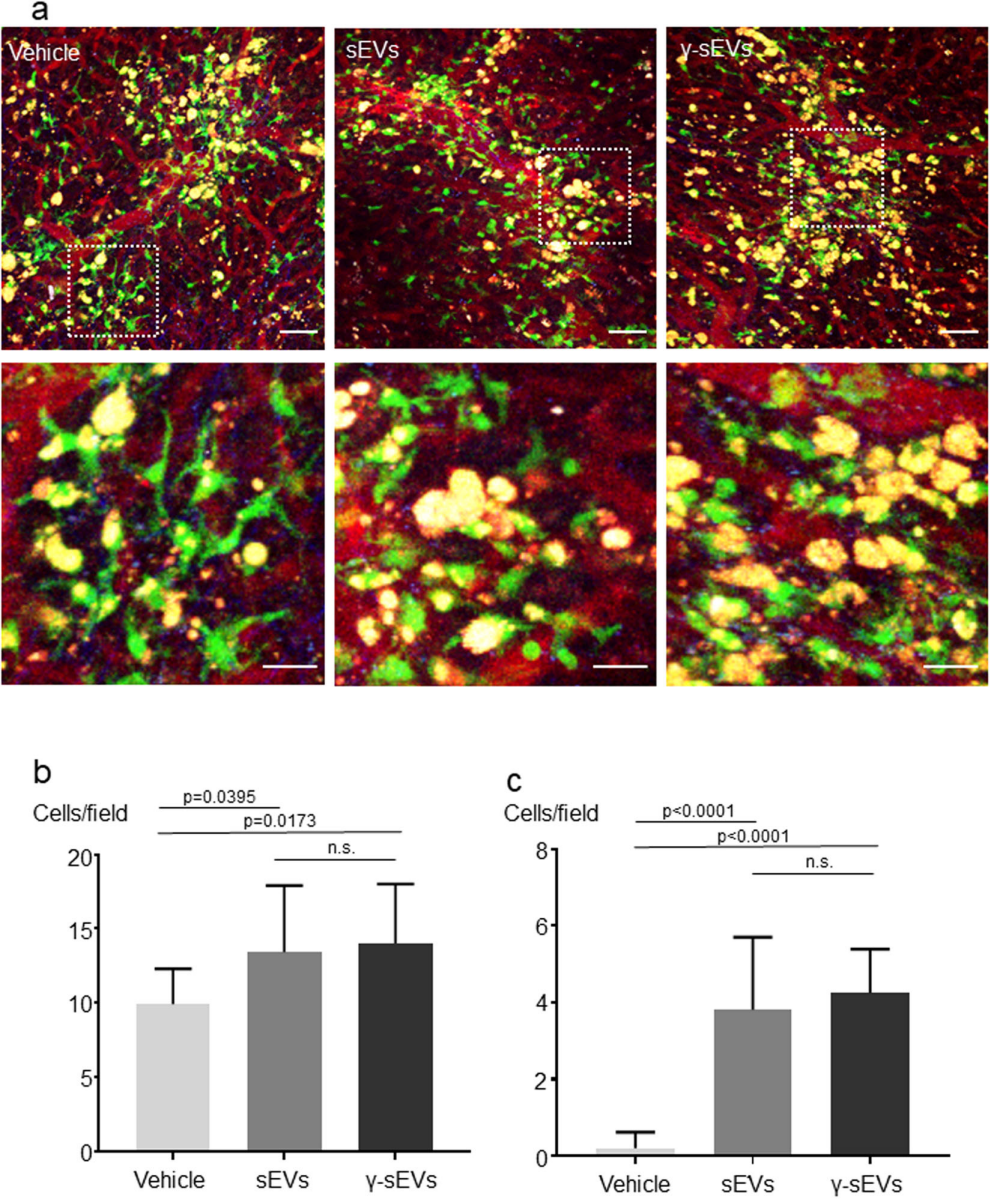
Intravital imaging after administering AD-MSC-sEVs (sEVs) or AD-MSC-γ-sEVs (γ-sEVs) in CCl4-damaged CX_3_CR1-EGFP mice. **a** Representative images 24 h after injecting the vehicle, sEVs, and γ-sEVs; scale bar = 50 μm (upper panels) and 20 μm (lower panels). (Supplementary Movie 3; vehicle, sEVs, and γ-sEVs from left to right in order). Lower panels: magnified view of upper panels. **b** Numbers of CX_3_CR1 + GFP-expressing cells gathering with or surrounding the hepatocyte debris. Data are presented as means ± SD; *n* = 10–11 per experiment. **c** Numbers of CX_3_CR1 + GFP-expressing cells migrating into the damaged area. Data are presented as means ± SD; *n* = 10–12 in each experiment.

In vitro analysis confirmed that sEVs enhance macrophage motility and phagocytic ability. Hence, we next analyzed the behavior of macrophages in vivo after injecting sEVs. To detect the macrophages in the liver, we used the CX_3_CR1-EGFP mouse. The number of CX_3_CR1-EGFP cells in healthy liver tissue was quite low; however, after CCl4 damage, the CX_3_CR1-EGFP cells accumulated at the damaged area^20^. Based on the single-cell RNA-sequencing results, CX_3_CR1+ cells were very similar to SAMs. Thus, we inferred that it was suitable to use this mouse to investigate the effect of sEVs on the behavior of SAMs. Liver cirrhosis was induced by 8 weeks of CCl4 administration in CX_3_CR1-EGFP mice, followed by administration of PBS (vehicle), 5 μg AD-MSC-sEVs, and 5 μg AD-MSC-γ-sEVs after 24 h. Intravital imaging was performed using two-photon excitation microscopy. We observed that the vehicle group differed from the sEV administration groups in three aspects. First, the shape of the CX_3_CR1-EGFP+ cells differed among groups. In the vehicle group, CX_3_CR1-EGFP+ cells were stable with an elongated extension; however, in the AD-MSC-sEV and AD-MSC-γ-sEV groups, the CX_3_CR1-EGFP+ cells were rounder in shape (Fig. 5a). Second, the relationship of CX_3_CR1-EGFP+ cells with hepatocyte debris was varied. We counted the numbers of CX_3_CR1-EGFP+ cells gathering and connecting with the comparable hepatocyte debris, and observed that in the AD-MSC-sEV (*p* = 0.0395) and AD-MSC-γ-sEV groups (*p* = 0.0173), more CX_3_CR1-EGFP+ cells were surrounded by hepatocyte debris than in the vehicle groups, thereby suggesting that tissue repair mechanisms were more active via the processing of cell debris (Fig. 5b). Third, the numbers of CX_3_CR1-EGFP+ migrating cells varied. There were significantly more migrating cells per unit time in the AD-MSC-sEV (*p* < 0.0001) and AD-MSC-γ-sEV (*p* < 0.0001) groups than in the vehicle groups (Fig. 5c). These results suggest that AD-MSC-derived sEVs affected the shape of the macrophages and effectively recruited them into the damaged area, which may have promoted tissue repair in the damaged liver.

## Discussion

**Fig. 6:**
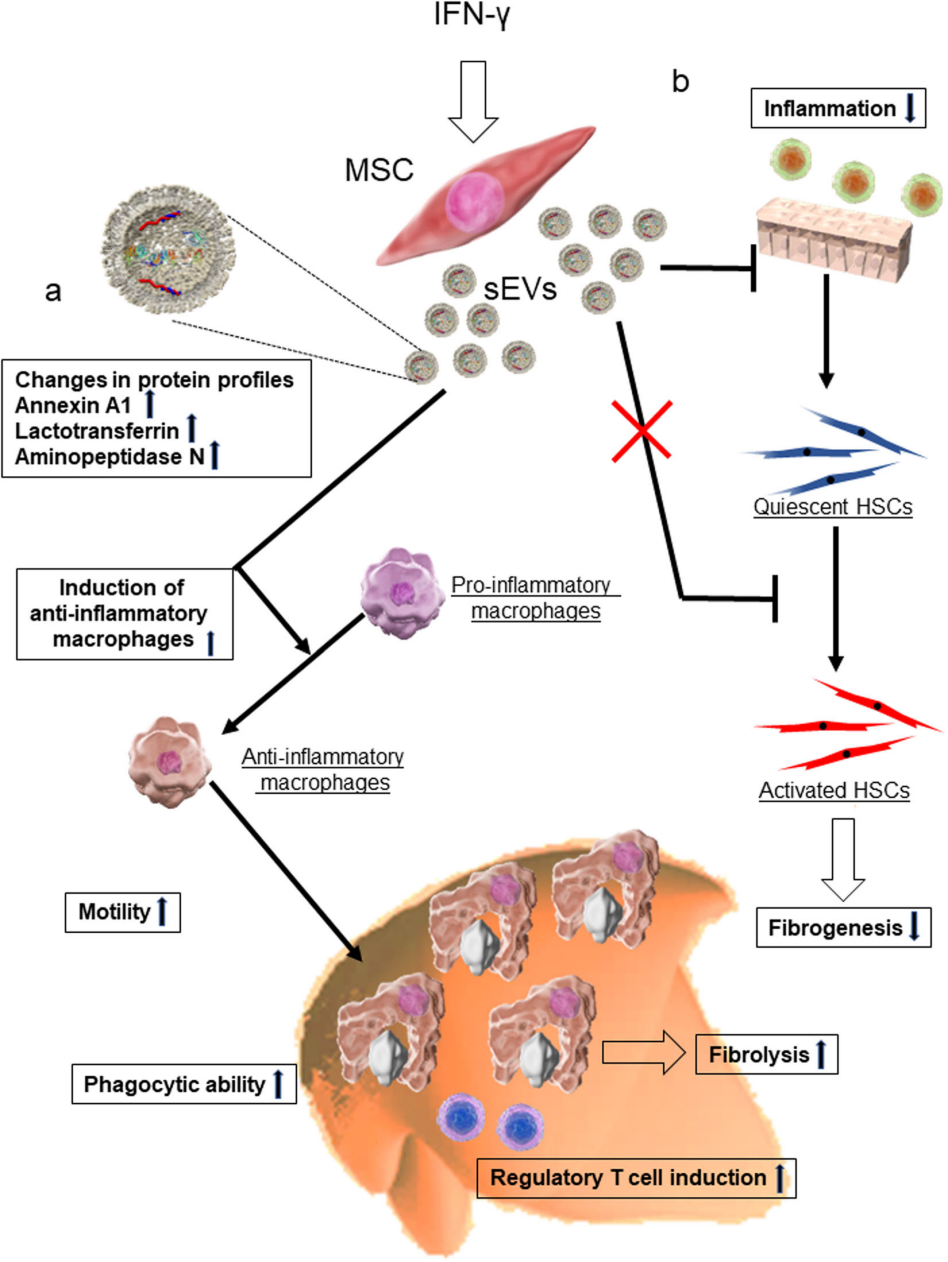
Schematic of the mechanisms underlying the amelioration of liver fibrosis by AD-MSC-γ-sEVs (γ-sEVs). **a** In γ-sEVs, the levels of proteins including annexin A1, lactotransferrin, and aminopeptidase N increased. These γ-sEVs induce the counts of anti-inflammatory macrophages with high motility and phagocytic ability, which increase repair of damaged tissue. In addition, these γ-sEVs induce regulatory T cell counts in the liver. These processes are related to fibrolysis. **b** The γ-sEVs also possess the ability to reduce inflammation; however, they do not prevent HSC activation, suggesting that γ-sEVs indirectly reduce fibrogenesis by reducing inflammation. HSCs, hepatic stellate cells.

In this study, we demonstrated that AD-MSC-γ-sEVs can induce anti-inflammatory macrophage counts, which are the key players in tissue repair, including the regression of fibrosis and promotion of tissue regeneration. Using up-to data imaging techniques, such as intravital imaging, these macrophages were shown to be highly motile and phagocytic in vitro and in vivo. While AD-MSC-γ-sEVs did not directly reduce HSC activation, which is important for fibrogenesis, they decreased inflammation, as indicated by the reduction in ALT levels and fibrolysis caused by macrophages; thus, AD-MSC-γ-sEVs showed potent therapeutic effects, which were superior to those of MSC therapy or AD-MSC-sEV therapy. We further analyzed the effects on the liver using single-cell RNA-seq after MSC or EV injection and showed that only γ-AD-MSCs and AD-MSC-γ-sEVs strongly induced the Treg cell counts. Our results clearly showed that AD-MSC-γ-sEVs perform multiple functions and are important means of communication between MSCs and immune cells, especially macrophages (Fig. 6). In addition, the proteome analysis revealed that the proteins of AD-MSC-γ-sEVs did not include IFN-γ, which confirms that administration of AD-MSC-γ-sEVs was not affected by IFN-γ contamination.

We also assessed the changes in the protein and miRNA levels in sEVs after IFN-γ pre-conditioning. In particular, we focused on five proteins based on published studies: annexin-A1, lactotransferrin, galectin-3-binding protein, lactadherin, and aminopeptidase N. We found that all these proteins are essentially related to macrophage polarization. Annexin- A1 is a multifunctional protein that can act both as an endogenous signaling molecule as well as a secreted mediator via formyl peptide receptors to promote macrophage polarization^21,22^. Annexin-A1 exhibits multidirectional anti-inflammatory effects in T cells, neutrophils, and macrophages. In particular, it can induce immunomodulatory effects and anti-inflammatory macrophages^23–25^. This protein is also related to the macrophage function of efferocytosis^26^. All these phenomena are consistent with our observations of macrophages in this study.

Lactotransferrin, also known as lactoferrin, is another important protein. It is a multifunctional iron-binding glycoprotein of the transferrin family detected in most mammalian exocrine secretions, as well as in neutrophil granules. It is known to sequester lipopolysaccharide (LPS) and pathogen-associated molecular patterns (PAMPs) and modulates endotoxin shock^27–29^. It is also known to increase myeloid-derived suppressor cell counts in newborn mice and regulatory T cells^30^. This protein likely induces Treg cell counts by AD-MSC-γ-sEV.

Aminopeptidase N, or CD13, is another multifunctional protein with three types of activities: peptide cleavage, endocytosis, and signaling. It is known to be involved in phagocytosis via dendritic cells and macrophages. In addition, IL-4, which is known to induce anti-inflammatory macrophages, induces CD13 and Fc epsilon RIIb expression, which may be dependent on the maturation stage of monocytes/macrophages^31,32^. This protein is highly expressed in AD-MSC-γ-sEVs; hence, we speculate that it also contributes to tissue repair.

Multifunctional lactadherin is also known as milk fat globule-EGF factor 8 (MFG-E8). It binds to apoptotic cells by recognizing phosphatidylserine, which enhances their engulfment by macrophages^33–35^. An et al. reported another function, in which the secretome of hepatocyte-like cells differentiated from umbilical cord tissue-derived MSC included MFG-E8, which suppressed transforming growth factor-β (TGF-β) and inhibited HSC activation^36^. In contrast, in our study, MFG-E8 expression was not changed by IFN-γ stimulation and AD-MSC-γ-sEVs did not inhibit HSC activation; however, the macrophages gained phagocytic ability both in vitro and in vivo after adding sEVs. Therefore, we conclude that MFG-E8 may be involved in inducing phagocytic ability.

Galectin-3-binding protein (LG3BP), also known as Mac-2-binding protein, is a multifunctional protein involved in innate immunity and host response to viral and bacterial infections. Its expression is induced by IFN-a, β, and γ and TNF-α. A previous study reported that LG3BP exhibits both immunosuppressive and immunostimulatory effects in vitro^37–40^. In our study, LG3BP did not induce CD206. Thus, this protein cannot strongly induce anti-inflammatory macrophages. Overall, AD-MSC-γ-sEVs harbor proteins that exert multidirectional effects, including induction of anti-inflammatory macrophages with phagocytic ability and Treg cells.

In contrast, we could not identify any role for the miRNAs in the AD-MSC-γ-sEVs. Among miRs, miR155, miR12, and miR21^41^ have been reported to induce anti-inflammatory macrophage counts; miR21 was highly expressed in AD-MSC-sEVs and AD-MSC-γ-sEVs, miR16^42^ was related to phagocytosis, and miR155^43^, miR181a, miR744^44^, and miR126a^45^ were implicated in the induction of Treg cell counts; however, their levels did not increase in our study. We did not identify any miRNAs that are induced by IFN-γ stimulation and contributed to the therapeutic effects on cirrhosis; however, unknown mechanisms due to combination of miRNAs may be elucidated in future studies.

Both MSC therapy and macrophage therapy have been reported to be effective. Our recent study using two-photon excitation microscopy revealed that most MSCs are trapped in the lung when MSCs were injected from the tail vain in CCl4 induced cirrhosis mouse model. We found that approximately 50% of administered BMMs migrated into hepatic lesions^9^. These MSC- and macrophage-based therapies used different cell sources; however, they exerted similar effects via macrophages, which induced regeneration of hepatocytes and biliary epithelial cells. It appears that cell-free MSC-sEV therapy is an attractive clinical option and that the effects of MSCs and MSC-sEVs are more wide-ranging than those of macrophages; however, the use of MSCs and MSC-sEVs to change the polarity of macrophages is an indirect method, and hence it is important to determine the safety and therapeutic effects of macrophage therapy, MSC therapy, and MSC-sEV therapy in the future. We believe that one of the key factors determining the success of MSC and MSC-sEV therapies is their ability to induce anti-inflammatory macrophages working for tissue repair. This should be evaluated in future studies. The Forbes group has begun a phase I autologous macrophage therapy and reported that primary outcomes of safety and feasibility have been met^12^. Whether direct injection of macrophages modified ex vivo is more effective than the polarity change of macrophages induced by injecting MSC-sEVs in vivo is an important issue that remains to be resolved. It is also possible that injection of both macrophages and MSC-sEVs be synergistic.

Using single-cell RNA-seq post-administration of MSCs or AD-MSC-sEVs, we demonstrated that both MSC and sEV therapies increased the abundance of anti-inflammatory macrophages, but only IFN-γ-pre-conditioned MSCs and sEVs increased the abundance of Treg cells. Recently, novel observations have been made using these methods. MacParland et al. analyzed the human hepatic immune microenvironment and showed the possibility of achieving more detailed analyses of macrophages using these methods than by using flow cytometry^46^. Ramachandran et al. further identified the cells present in the fibrotic niche of the liver, including the TREM2+ CD9+ subpopulation of macrophages, SAMs, as well as ACKR1+ and PLVAP+ endothelial cells^19^. Application of single-cell analysis following cell and sEV therapy will be important for analyzing the mechanisms underlying this therapy.

Clearly, sEVs are attractive diagnostic and therapeutic tools not only in the field of oncology, but also in regenerative medicine. In our system, the therapeutic effect of sEVs was not inferior to that of MSCs^16,47,48^. In fact, MSC-sEV therapy is being considered worldwide for its several merits. While most MSCs administered via the peripheral vein are trapped in the lungs, theoretically, they can migrate to the target organ more easily. Wiklander et al. reported that following systemic injection of sEVs derived from HEK293T (human embryonic kidney) cells, the liver showed the most uptake, followed by the spleen and gastrointestinal tract^49^. Imai et al. reported that B16BL6-derived sEVs injected via the tail vein were incorporated by macrophages in the liver, most of which cleared from the blood within several hours. In contrast, when macrophages were depleted, the sEVs persisted in the blood for a longer duration^50^. These results suggest that MSC-sEV is an ideal drug delivery system for treating liver diseases. However, this hypothesis must be verified in the future.

In the clinical settings, a strategy that addresses multiple aspects of sEVs, such as quantity, selectivity, and content should be designed to improve the performance of MSC-sEV therapy for liver disease. Large quantities of sEVs would need to be prepared to address the quantity issue. Primary cells or cells immortalized using hTERT or c-myc may be used for this purpose. Studies have shown that gene expression profiles can be altered substantially by immortalization; however, these changes may not be manifested in the secreted sEVs. Rohde et al. reported that the use of hTERT resulted in a comparatively stable gene expression profile for relevant cell surface markers. The protein and miRNA profiles in immortalized cells must be assessed, as these are likely to affect therapeutic potential. Enrichment of sEVs is another challenge that must be overcome. They also reported that tangential flow filtration combined with ultracentrifugation is an efficient, scalable, and rapid method for sEVs enrichment^51^. Innovations that improve the efficiency of sEV collection will be required to expand the scope of sEV therapy. To address the selectivity issue, multiple endocytic pathways, including clathrin-dependent endocytosis and clathrin-independent pathways, such as caveolin-mediated uptake, micropinocytosis, phagocytosis, and lipid raft-mediated internalization, can be utilized^52^. The possibility that the proteins and glycoproteins expressed on the surface of sEV may determine selectivity has been reported^53^. Whether IFN-γ stimulation changes the cell surface markers of the sEVs was not investigated in this study, which is a major limitation; hence, modulation of surface proteins and/or glycoproteins should be investigated in the future. With respect to content, we showed that after IFN-γ stimulation, protein profiles of sEVs changed significantly, which affected therapeutic efficacy; thus, pre-conditioning” can be used to change the content of sEVs.

Kamerkar et al. reported the development of the “iExosome,” which can improve the therapeutic efficacy for pancreatic cancer. First, they selected CD47-expressing exosomes. CD47 can protect exosomes from phagocytosis by monocytes or macrophages, thereby enhancing their retention in the blood. Second, they engineered exosomes to harbor siRNA or shRNA specific for oncogenic KRASG12D, which is the key driver of pancreatic cancer^54^. They also developed a strategy for bioreactor-based large-scale production of clinical grade “iExosomes” using GMP standards and are currently starting a clinical study^55^.

There are additional limitations of this study. We could not detect the sEVs after uptake in vitro and in vivo due to their small size and did not analyze the role of miRNAs in detail. We did not determine the mechanisms underlying Treg cell count induction by IFN-γ pre-conditioned MSCs and sEVs. In addition, not all macrophages in the liver were CX_3_CR1+. Nonetheless, our results provide credible evidence that in addition to macrophage therapy, IFN-γ pre-conditioned sEVs are an attractive strategy for treating liver diseases.

## Methods

### Mice

C57BL/6 male mice were purchased from Charles River (Yokohama, Japan). CX_3_CR1-enhanced green fluorescent protein (EGFP) knock-in mice were purchased from the Jackson Laboratory (Bar Harbor, ME, USA). GFP-transgenic mice (Tg(CAG-EGFP)C14-Y01-FM131Osb) were kindly provided by Professor Masahito Ikawa, Osaka University. DsRed-transgenic mice (Tg(CAG-DsRedMST1)Nagy/J) were purchased from the Jackson Laboratory. Mice were housed in a specific pathogen-free environment and maintained under standard conditions with a 12-h day/night cycle and had ad libitum access to food and water. All animal experiments were conducted in compliance with the regulations of Niigata University and Osaka University and were approved by both institutions’ Institutional Animal Care Committees.

### Human MSCs

Human adipose tissue-derived MSCs (AD-MSCs; passage 2) were obtained from PromoCell (Heidelberg, Germany; catalog number C-12977) and expanded until passage 4 using StemPro MSC SFM XenoFree medium (Thermo Fisher Scientific, Waltham, MA, USA) in low oxygen (5% O_2_) in the presence of 5% CO_2_ at 37 °C. Cells were tested by PromoCell for morphology, proliferation potential, adherence rate, and viability. Cells were analyzed by flow cytometry using a comprehensive panel of markers, CD73/CD90/CD105 and CD14/CD19/CD34/CD45/HLA-DR. Adipogenic, osteogenic, and chrondrogenic differentiation assays were performed for each lot in the absence of antibiotics and antimycotics.

### Pre-conditioning of AD-MSCs

Passage 4 AD-MSCs were cultured to 70% confluence in the presence of 5% O_2_ at 37 °C and 100 ng/ml human recombinant IFN-γ (R & D Systems, Minneapolis, MN, USA) was added before culture for 48 h. IFN-γ-pre-conditioned AD-MSCs (γ-AD-MSC) were used for injection and the supernatant was used for collection of sEVs.

### Collection of sEVs

Passage 4 AD-MSC or γ-AD-MSC were cultured without serum in advanced Dulbecco’s modified Eagle’s medium (DMEM) (Thermo Fisher Scientific) with GlutaMAX^TM^ (100x) (Thermo Fisher Scientific) for 48 h. The supernatant was collected and centrifuged at 2000 *g* for 10 min to remove cell debris and filtered using a 0.22 μm filter (Stericup^TM^ Quick Release Durapore^TM^, Merck Millipore, Burlington, MA, USA). The filtered supernatant was ultracentrifuged at 210,000 *g* for 70 min at 4 °C using Optima XL-100K (Beckman Coulter, Inc, Brea, CA, USA) in a swing rotor SW41Ti (Beckman Coulter, Inc). The residual fraction was washed with phosphate buffered saline (PBS; pH 7.4) and again collected by ultracentrifugation at 210,000 *g*. PBS was added to the final sEV-enriched fraction. Protein was quantified using Qubit3 (Thermo Fisher Scientific).

### Nanoparticle tracking

After sEV isolation, particle concentration was measured using a Nanosight LM10HS equipped with a blue laser system (Malvern Instruments Ltd., Malvern, UK). Samples were diluted 25-fold with PBS. Particles were visualized by light scattering using a conventional optical microscope aligned perpendicularly to the beam axis. Data were analyzed using NTA software (Malvern Instruments Ltd.) with a detection threshold set to 7 and temperature approximately 25 °C. The Brownian motion of each particle was tracked between frames to calculate its size using the Stokes–Einstein equation. Each sample was measured three times and averaged values were used for the statistical analysis.

### Western blot

Lysates of sEVs were extracted by M-PER (Thermo Fisher Scientific). Total proteins were separated by SDS-PAGE using 4–20% Novex 4–20% Tris-Glycine Mini Gels (Thermo Fisher Scientific) and transferred to PVDF membranes (Thermo Fisher Scientific). Electrophoresis, blotting, and antibody treatment were performed using a Mini Gel Tank (Thermo Fisher Scientific), Pierce Power Blotter Stainer System (Thermo Fisher Scientific), and iBind Western Systems following the manufacturer’s instructions. Primary antibodies (Exosome-anti CD9; 10626D; dilution: 1:50, CD63; 10628D; dilution: 1:50, and CD81; 10630D; dilution: 1:50 for western blot) were purchased from Thermo Fisher Scientific and secondary antibodies (anti-mouse IgG HRP-linked whole antibody) were purchased from GE Healthcare (Chicago, IL, USA) (Supplementary Fig. 1) All blots derive from the same experiment and were processed in parallel.

### Macrophage culture

Bone marrow cells collected from mouse femurs were cultured at 37 °C in the presence of 5% CO_2_ in ultra-low attachment flasks (Corning, Corning, NY, USA) and medium (DMEM/F12; Thermo Fisher Scientific) containing 20 ng/ml colony stimulating factor-1 (CSF-1) (Peprotech, Rocky Hill, NJ, USA); the medium was changed twice weekly as described previously^9^. After 5 or 7 days, the collected macrophages were harvested.

### Macrophage polarity assay

Macrophages were seeded in 6-well ultra-low attachment dishes (Corning) at 5 × 10^6^ cells/well, followed by the addition of sEVs (final concentration 100 ng/ml) or recombinant proteins. Recombinant human annexin A1 (Abcam, Cambridge, UK; final concentration 100 ng/ml), recombinant galectin-3 binding protein (LG3BP) (Aviva Systems Biology, San Diego, CA, USA; final concentration 1 ng/ml), recombinant human lactoferrin (lactotransferrin) (Abcam; final concentration 0.1 ng/ml), recombinant human lactadherin (MFGE8) (R & D Systems, Inc.; final concentration 100 ng/ml), and recombinant human aminopeptidase N/CD13 (R & D Systems; final concentration 100 ng/ml) were used. After 48 h, macrophages were harvested and mRNA expression levels of genes encoding pro- (*Il-6, Tnf-a, Mcp-1, Inos*) and anti- (*Il-10, Ym-1, Fizz-1, Cd206*) inflammatory factors were evaluated using real-time PCR.

### Macrophage motility assay

GFP+ cultured macrophages were seeded into 24-well microplates (AGC Techno Glass Co., Ltd., Haibara, Japan) and incubated for 30 min at 37 °C in a 5% CO_2_ incubator. Then the vehicle (PBS) or sEVs (final concentration; 5 μg/ml) were added and incubated for 240 min. After incubation, the cells were observed every 30 s using a confocal microscope (A1; Nikon, Tokyo, Japan) with a 488 nm excitation wavelength.

### Macrophage phagocytosis assay

DsRed+ cultured macrophages were seeded and incubated as described above for the motility assay. Vehicle (PBS) or sEVs (final concentration; 5 μg/ml) were added to the cells for 120 min. After incubation, 10 μl pHrodo™ Green Zymosan Bioparticles conjugate for phagocytosis (0.5 mg/ml) (Thermo Fisher Scientific) was added to each well and cells were imaged every 30 s for 90 min using a confocal microscope (A1; Nikon, Tokyo, Japan) with 488 or 561-nm laser illumination. The phagocytosing macrophages were counted at 30, 60 and 90 min after particles were added.

### MSC and sEV injection into cirrhotic mice

Male mice were intraperitoneally injected twice weekly with 1.0 ml/kg carbon tetrachloride (CCl4; Wako Pure Chemical Industries Ltd., Osaka, Japan) dissolved in corn oil (Wako Pure Chemical Industries Ltd., 1:10 v/v) to induce cirrhosis. Eight weeks after the CCl4 injection, PBS (vehicle), AD-MSCs (1 × 10^6^ cells/mouse), IFN-γ pre-conditioned AD-MSCs (γ-AD-MSC; 1 × 10^6^ cells/mouse), adipose tissue-derived MSC-sEVs (AD-MSC-sEVs) (2 μg, 5 μg/ mouse), or AD-MSC-γ-sEVs (2 μg, 5 μg/ mouse) were injected into the tail vein. Serum and liver fibrosis analyses were performed 4 weeks after the injection.

### Real-time polymerase chain reaction (PCR)

Total RNA was extracted using TRIzol reagent (Thermo Fisher Scientific) or the RNeasy kit (Qiagen, Venlo, Netherlands) and reverse-transcribed using the QuantiTect reverse transcription kit (Qiagen) as per the manufacturer’s protocol. Gene expression analysis was performed using pre-validated QuantiTect primers (Supplementary Table 2) and QuantiTect SYBR reagent (Qiagen) or SYBR Premix EX TaqII (Takara Bio, Kusatsu, Japan). Real-time PCR was conducted on a Step One Plus system (Applied Biosystems, Foster City, CA, USA). The amplification steps included denaturation at 95 °C for 15 min, followed by 40 cycles of denaturation at 95 °C for 15 s, annealing at 65 °C for 30 s, and extension at 72 °C for 30 s. Data were obtained from at least three independent samples. *Gapdh* and *Actb1* were used as internal controls. The fold-change in relative gene expression from the control was calculated using the 2^–ΔΔCt^ method.

### Culture of mouse hepatic stellate cells (HSCs)

HSCs were isolated from the livers of 9-month-old male mice. After isolation of the non-parenchymal cell (NPC) fraction using a two-step collagenase perfusion method, NPCs were subjected to density centrifugation using 11% Histodenz (Sigma-Aldrich, Saint Louis, MO, USA). After isolation, mouse HSCs were cultured on Cellmatrix Type I-C (Nitta gelatin, Yao, Japan) coated 24 well plates at a density of 30,000 cells/cm^2^ in DMEM (Sigma) supplemented with 10% FBS (Thermo Fisher Scientific), non-essential amino acid solution (Thermo Fisher Scientific), and penicillin-streptomycin-glutamine (Thermo Fisher Scientific). After 24 h of culture, cells were washed with PBS, the medium was changed, and AD-MSC-sEVs or AD-MSC-γ-sEVs were added to each well at a final concentration of 2.25 or 0.025 mg/well. Cells were cultured for 4 days in a 5% CO_2_-ambient O_2_ environment.

### Proteomics of sEVs

The sEV samples were added to a 100 µl reduction processing solution [100 mM NH_4_HCO_3_ containing 0.15% (w/v) dithiothreitol as the reducing agent] and incubated at 57 °C for 30 min. After incubation, 100 µl alkylation process solution [100 mM NH_4_HCO_3_ containing 1% (w/v) iodoacetamide as the alkylation reagent] was added and the mixture was incubated at 20 °C. After adding 100 µl modified trypsin and 50 mM NH_4_HCO_3_ (in this order), the mixture was incubated overnight at 30 °C. Digestive fluid was dried using a centrifugal concentrator (CC–105, TOMY SEIKO, Tokyo, Japan), dissolved in 30 µl 0.1% formic acid, and centrifuged at 20,000 *g* for 10 min. The supernatant was used for nano-liquid chromatography-mass spectrometry (LC-MS/MS) (Ultimate 3000: Dionex, Sunnyvale, CA, USA connected to QExactive Orbitrap: Thermo Fisher Scientific, Bremen). The samples were loaded on the column (75 µm internal diameter and 500 mm length: L-column2 ODS (octadecylsilane), CERI, Tokyo, Japan) using nano-high performance liquid chromatography (UltiMate 3000, Dionex Sunnyvale, CA, USA). Eluted peptides were analyzed using Q-Exactive Plus MS (Thermo Fisher Scientific). Nano-LC and MS were controlled by Xcalibur (Thermo Fisher Scientific). To identify and quantify proteins, the nano-LC-MS/MS spectra were analyzed using the Mascot server (version 2.5.1, Matrix Science Inc, MA, USA). The spectral data were submitted to SWISS–PROT and National Center of Biotechnology Information (NCBI) of green plants. The Mascot search parameters were: threshold of the ion score cut–off, 10; peptide tolerance, 0.8 Da; MS/MS tolerance, 0.5 Da; peptide charge 2-4, trypsin as the enzyme and allowing up to two missed cleavages; carbamidomethylation of cysteines as a fixed modification; and oxidation of methionine as a variable modification. Statistical analysis of protein spectral counts was performed using Scaffold (version 4.10.0, Proteome Software Inc., OR, USA). Significant differences were detected using Fisher’s least significant difference procedure at *p* < 0.05. Only identified proteins with a false discovery rate (FDR) of less than 0.05 were considered.

### Analysis of sEV miRNA content

Following sEV collection, miRNA extraction, library preparation, sequencing, mapping, and gene expression analyses were performed using DNAFORM (Yokohama, Kanagawa, Japan). Extraction of miRNAs was performed using the miRNeasy serum/plasma kit (Qiagen, Hilden, Germany). The quality and quantity of the extracted miRNAs were assessed using the QuantiFluor RNA system (Promega, Madison, WI) and the Agilent Small RNA kit of the BioAnalyzer 2100 system (Agilent Technologies, Santa Clara, CA, USA). The miRNA-seq library was prepared using the QIAseq miRNA library kit (Qiagen) following the manufacturer’s instructions. Libraries were sequenced using a NextSeq 500 (Illumina, San Diego, CA, USA) to generate 75 nucleotide single reads. Duplicated reads were removed from raw reads using seqkit (ver. 0.10.1), and adapter sequences were trimmed using cutadapt (ver. 1.16). The trimmed reads were mapped to the human and mouse genomes using the GRCh38.p13 and GRCm38.p6 genome assemblies, respectively, using STAR (ver. 2.7.2b) with the following parameters: --alignEndsType EndToEnd --outFilterMismatchNmax 1 --outFilterMultimapScoreRange 0 --outReadsUnmapped None --outSAMtype BAM Unsorted --outFilterMultimapNmax 10 --outSAMunmapped Within --outFilterScoreMinOverLread 0 --outFilterMatchNminOverLread 0 --outFilterMatchNmin 16 --alignSJDBoverhangMin 1000 --alignIntronMax 1. Reads mapped to miRNA genes were quantified using featureCounts of the Subread package (ver. 1.6.1) with the following parameters: -s 1 -O -M -p -C -B -T. Differential gene expression was performed using DESeq2 (ver. 1.18.1).

### Serum analyses

Blood samples were obtained from the abdominal aorta of mice 4 weeks after cell or sEV injection. Serum alanine aminotransferase (ALT), alkaline phosphatase (ALP), and albumin (ALB) concentrations were determined by Oriental Yeast Co., ltd. (Tokyo, Japan).

### Sirius red staining

To quantify fibrosis, liver tissues were fixed with 10% formalin 4 weeks after cell or sEV injection. The fixed tissue was cut into 4-μm-thick sections and stained with Sirius Red. Ten images were randomly captured from each section and measured using ImageJ software version 1.6.0 20 (National Institutes of Health, Bethesda, MD, USA).

### Hydroxyproline assay

Four weeks after cell or sEV administration, cirrhotic livers were used to measure the concentration of the collagen component hydroxyproline. Liver samples (20 mg) were homogenized and subjected to a QuickZyme hydroxyproline assay (QuickZyme Bioscience, Zernikedreef, Netherlands) according to the manufacturer’s protocol.

### Immunostaining

For immunohistochemistry, tissues were fixed in 10% formalin and cut into 4 μm sections. Sections were subjected to antigen retrieval by heating the sample by microwave in a 10 mM sodium citrate buffer (pH 6.0). Four weeks after cell or sEV administration, the cirrhotic livers were stained using an anti-αSMA antibody (Ab124964, Abcam, Cambridge, UK; dilution: 1:200). Slides were then stained using the Vectastain® ABC kit (Vector Laboratories, Inc. Burlingame, CA, USA) and the DAB substrate (Muto Pure Chemicals, Tokyo, Japan). For αSMA immunocytochemistry, cultured cells were fixed with methanol and stained with an anti-αSMA antibody (Ab124964, Abcam; dilution: 1:200) followed by incubation with a donkey anti-rabbit secondary antibody (Alexa Fluor Plus 594, A48254, Thermo Fisher Scientific; dilution: 1:400), and then mounted using VECTASHIELD Mounting Medium with DAPI (Vector Laboratories).

### Intravital imaging

Mice were anesthetized with isoflurane (Wako Pure Chemical Industries, Ltd.), the median lobe of the liver was surgically exposed, and the internal surface was observed using inverted two-photon excitation microscopy (A1R-MP, Nikon, Tokyo, Japan) driven by a laser (Chameleon Vision Ti: Sapphire, Coherent) tuned to 880 nm, with a water multi-immersion objective lens (Plan Fluor N.A., 0.75; Nikon). Emissions were detected via bandpass emission filters at 525/50 nm (EGFP) and 575/25 nm (DsRed). Raw imaging data were processed using Imaris software (Bitplane, Zurich, Switzerland). To analyze the relationship between hepatocyte debris and macrophages, circles of 100-μm diameter were drawn to include hepatocyte debris that occupied approximately a quarter of the circle, followed by counting of the number of CX_3_CR1+ EGFP-expressing cells in video recordings. Images were divided into four areas and CX_3_CR1+ EGFP-expressing cells that migrated into the damaged area were counted for 15 minutes.

### Image analysis to track morphological change in macrophage

Cell shapes were determined and tracked by the NIS Elements image analysis software (Nikon) as previously described. We defined three distinct areas: at the initial timeframe (*t* = 0) (A); at the final timeframe (*t* = 2) (C); and the overlap between these two time frames (B). The cell deformation index (CDI) was calculated as (A + C) / (A + B), representing the ratio of area change over the 2 min divided by that of the previous time frame^56^.

### Single-cell transcriptome analysis

After 8 weeks of CCl4-induced liver damage, PBS (vehicle), AD-MSCs (1 × 10^6^ cells/mouse), γ-AD-MSCs (1 × 10^6^ cells/mouse), AD-MSC-sEVs (5 μg/mouse), or AD-MSC-γ-sEVs (5 μg/mouse) were injected into the tail veins of mice. Forty-eight hours after injection, mice were euthanized, cirrhotic livers were perfused with PBS, and then harvested and dissociated into single cells using the liver dissociation kit (Miltenyi Biotec, Bergisch Gladbach, Germany) according to the manufacturer’s protocol. The dissociated cells were sorted using Mofl XDP (Beckman Coulter) after propidium iodide (PI) staining (1 µg/ml) to exclude dead cells. Cells were captured on the 10x Genomics Chromium controller (10x Genomics, Pleasanton, CA, USA) and sequencing libraries were prepared according to the Chromium single cell 3′ reagent kit V2 user guide (10x Genomics PN-120237). Libraries were sequenced using an Illumina HiSeq 2500 (Illumina; San Diego, CA, USA) and analyzed using the 10x cellranger pipeline (ver. 3.0.2) and Seurat (ver3.0).

### ELISA and Griess assay

ELISA for IL-6, MCP-1, and TNF-α was performed using the supernatant collected from the macrophage culture 3 days after addition of sEVs or proteins, using the IL-6 mouse ELISA Kit (Thermo Fisher Scientific), the MCP-1 mouse ELISA Kit (Thermo Fisher Scientific), and the TNF-α mouse ELISA Kit (Thermo Fisher Scientific), respectively, according to the manufacturer’s protocol. The Griess assay was performed using the Nitrite assay kit (Bio Vision, Milpitas, CA, USA) according to the manufacturer’s protocol.

### Statistical analyses

Data were processed using GraphPad Prism v. 7 (GraphPad Software Inc., La Jolla, CA, USA) and are presented as means ± SD. Data were assessed using the Mann‒Whitney test and differences between groups were analyzed using one-way analysis of variance (ANOVA). Differences were considered significant at *p* < 0.05.

### Reporting summary

Further information on research design is available in the Nature Research Reporting Summary linked to this article.

## Supplementary information

Supplementary material

Reporting Summary Checklist

Supplemental Video 1

Supplemental Video 2

Supplemental Video 3

## Data Availability

The data that support the findings of this study are available from the corresponding author upon reasonable request. The proteomics data are available via ProteomeXchange with identifier PXD024647. The miRNA and single cell transcriptome analysis data have been deposited in the GEO public database repository (accession number GSE167863 and GSE168042).
