# Supplementary material for: Small extracellular vesicles derived from interferon-γ pre-conditioned mesenchymal stromal cells effectively treat liver fibrosis
Source: NPJ Regen Med. 2021 Mar 30;6:19. doi: 10.1038/s41536-021-00132-4 (PMC8010072; doi:10.1038/s41536-021-00132-4)
Supplement: Supplementary file 1 — Supplementary material [file 41536_2021_132_MOESM1_ESM.pdf]

Supplementary Table 1. Proteomic analysis of AD-MSC-sEVs (sEVs) or AD-MSC-γ-sEVs (γ-sEVs)

Identified Proteins	Accession Num	MW	Fisher's Exact Test	Fold Change	sEVs	γ-sEVs
Collagen alpha-3(VI) chain	CO6A3_HUMAN	344 kDa	< 0.00010	0.8	1600	1233
Fibronectin	FINC_HUMAN	263 kDa	< 0.00010	0.6	499	319
Collagen alpha-1(VI) chain	CO6A1_HUMAN	109 kDa	0.041	0.8	424	341
Collagen alpha-2(VI) chain	CO6A2_HUMAN	109 kDa	0.017	0.7	212	154
Serum albumin	ALBU_HUMAN	69 kDa	< 0.00010	1.5	138	209
Keratin, type II cytoskeletal 1	K2C1_HUMAN	66 kDa	0.2	1.1	65	69
Haptoglobin	HPT_HUMAN	45 kDa	0.0032	1.6	43	67
Hemopexin	HEMO_HUMAN	52 kDa	< 0.00010	3.1	25	77
Keratin, type I cytoskeletal 10	K1C10_HUMAN	59 kDa	0.41	0.8	45	38
Aminopeptidase N	AMPN_HUMAN	110 kDa	0.00023	2.4	18	43
Serotransferrin	TRFE_HUMAN	77 kDa	0.00029	2.4	17	41
Collagen alpha-2(I) chain	CO1A2_HUMAN	129 kDa	0.22	1.2	21	25
Actin, cytoplasmic 1	ACTB_HUMAN	42 kDa	0.27	1.2	19	22
Keratin, type II cytoskeletal 2 epidermal	K22E_HUMAN	65 kDa	0.15	0.6	23	14
Afamin	AFAM_HUMAN	69 kDa	< 0.00010	4.8	5	24
Hemoglobin subunit alpha	HBA_HUMAN	15 kDa	0.4	1.1	12	13
Transforming growth factor-beta-induced protein ig-h3	BGH3_HUMAN	75 kDa	0.17	0.6	20	12
Tenascin	TENA_HUMAN	241 kDa	0.042	2.1	7	15
Alpha-1B-glycoprotein	A1BG_HUMAN	54 kDa	0.0053	3.8	4	15
Histone H2A type 1-B/E	H2A1B_HUMAN	14 kDa	0.006	0.3	21	6
Keratin, type I cytoskeletal 9	K1C9_HUMAN	62 kDa	0.26	1.2	13	16
Dynein heavy chain 3, axonemal	DYH3_HUMAN	471 kDa	0.075	0	4	0
Versican core protein	CSPG2_HUMAN	373 kDa	0.09	1.9	7	13
Lactadherin	MFGM_HUMAN	43 kDa	0.4	1.1	12	13
Basement membrane-specific heparan sulfate proteoglycan core protein	PGBM_HUMAN	469 kDa	0.44	0.7	6	4
Lactotransferrin	TRFL_HUMAN	78 kDa	< 0.00010	INF	0	24
Trypsin-2	TRY2_HUMAN	26 kDa	0.028	0.2	10	2
BPI fold-containing family A member 1	BPIA1_HUMAN	27 kDa	< 0.00010	INF	0	17
Collagen alpha-1(I) chain	CO1A1_HUMAN	139 kDa	0.33	1.3	7	9
Histone H4	H4_HUMAN	11 kDa	0.015	0.3	17	5
Alpha-2-macroglobulin	A2MG_HUMAN	163 kDa	0.24	1.5	6	9
Polymeric immunoglobulin receptor	PIGR_HUMAN	83 kDa	< 0.00010	INF	0	17
5'-nucleotidase	5NTD_HUMAN	63 kDa	0.23	1.6	5	8
Annexin A2	ANXA2_HUMAN	39 kDa	0.33	1.3	7	9
Integrin beta-1	ITB1_HUMAN	88 kDa	0.0055	INF	0	7
Elongation factor 1-alpha 1	EF1A1_HUMAN	50 kDa	0.43	1.2	6	7
Galectin-3-binding protein	LG3BP_HUMAN	65 kDa	0.0021	6.5	2	13
HLA class I histocompatibility antigen, B-49 alpha chain	1B49_HUMAN	41 kDa	< 0.00010	INF	0	15
Heat shock 70 kDa protein 1A	HS71A_HUMAN	70 kDa	0.0057	0	8	0
Hemoglobin subunit beta	HBB_HUMAN	16 kDa	0.31	1.7	3	5
Immunoglobulin heavy constant alpha 1	IGHA1_HUMAN	38 kDa	0.00059	INF	0	10
Annexin A6	ANXA6_HUMAN	76 kDa	0.44	0.7	6	4
BPI fold-containing family B member 1	BPIB1_HUMAN	52 kDa	0.0012	INF	0	9
Major vault protein	MVP_HUMAN	99 kDa	0.0055	INF	0	7
Annexin A5	ANXA5_HUMAN	36 kDa	0.011	0	7	0
Annexin A1	ANXA1_HUMAN	39 kDa	0.0026	INF	0	8
Pecanex-like protein 3	PCX3_HUMAN	222 kDa	0.23	INF	0	2
Immunoglobulin kappa constant	IGKC_HUMAN	12 kDa	0.0012	INF	0	9
Histone H2B type F-S	H2BFS_HUMAN	14 kDa	0.04	0	5	0
Ubiquitin-60S ribosomal protein L40	RL40_HUMAN	15 kDa	0.56	1	5	5
Glyceraldehyde-3-phosphate dehydrogenase	G3P_HUMAN	36 kDa	0.27	0.4	5	2
Pyruvate kinase PKM	KPYM_HUMAN	58 kDa	0.61	1	3	3
Periostin	POSTN_HUMAN	93 kDa	0.42	0.6	5	3
Alpha-2-HS-glycoprotein	FETUA_HUMAN	39 kDa	0.012	INF	0	6
Keratin, type II cytoskeletal 6C	K2C6C_HUMAN	60 kDa	0.024	INF	0	5
Integrin alpha-2	ITA2_HUMAN	129 kDa	0.051	INF	0	4
Fatty acid synthase	FAS_HUMAN	273 kDa	0.27	0	2	0
Protein S100-A9	S10A9_HUMAN	13 kDa	0.012	INF	0	6
Deleted in malignant brain tumors 1 protein	DMBT1_HUMAN	261 kDa	0.051	INF	0	4
E3 ubiquitin-protein ligase SHPRH	SHPRH_HUMAN	193 kDa	0.14	0	3	0
Alpha-enolase	ENOA_HUMAN	47 kDa	0.11	INF	0	3
N-acetylmuramoyl-L-alanine amidase	PGRP2_HUMAN	62 kDa	0.23	INF	0	2
Keratin, type I cytoskeletal 16	K1C16_HUMAN	51 kDa	0.051	INF	0	4
Vitamin D-binding protein	VTDB_HUMAN	53 kDa	0.024	INF	0	5
Tubulin alpha-1B chain	TBA1B_HUMAN	50 kDa	0.11	INF	0	3
Serine protease HTRA1	HTRA1_HUMAN	51 kDa	0.024	INF	0	5
Protein S100-A8	S10A8_HUMAN	11 kDa	0.024	INF	0	5
Programmed cell death 6-interacting protein	PDC6I_HUMAN	96 kDa	0.27	0	2	0
Collagen alpha-1(III) chain	CO3A1_HUMAN	139 kDa	0.27	0	2	0
Platelet-derived growth factor receptor beta	PGFRB_HUMAN	124 kDa	0.23	INF	0	2
Leucine-rich alpha-2-glycoprotein	A2GL_HUMAN	38 kDa	0.051	INF	0	4
Immunoglobulin gamma-1 heavy chain	IGG1_HUMAN	49 kDa	0.11	INF	0	3
Clusterin	CLUS_HUMAN	52 kDa	0.11	INF	0	3
Tryptophan--tRNA ligase, cytoplasmic	SYWC_HUMAN	53 kDa	0.23	INF	0	2
Plasma protease C1 inhibitor	IC1_HUMAN	55 kDa	0.23	INF	0	2
Prolyl endopeptidase FAP	SEPR_HUMAN	88 kDa	0.23	INF	0	2
Immunoglobulin lambda-1 light chain	IGL1_HUMAN	23 kDa	0.23	INF	0	2

Supplementary Table 2. List of primers used for real-time PCR

Supplemental Table 1			
List of primers of real time PCR			
Primer	Catalog number	Species	Company
Gapdh	QT01658692	Mouse	Qiagen
Pro-inflammatory macrophage factors			
Il-6	QT00098875	Mouse	Qiagen
Tnf-a	QT00104006	Mouse	Qiagen
Inos	QT01547980	Mouse	Qiagen
Mcp-1	QT00167832	Mouse	Qiagen
Anti-inflammatory macrophage factors			
Il-10	QT00106169	Mouse	Qiagen
Ym-1	QT00108829	Mouse	Qiagen
Fizz-1	QT00254359	Mouse	Qiagen
Cd206	QT00103012	Mouse	Qiagen

Supplementary Figure 1

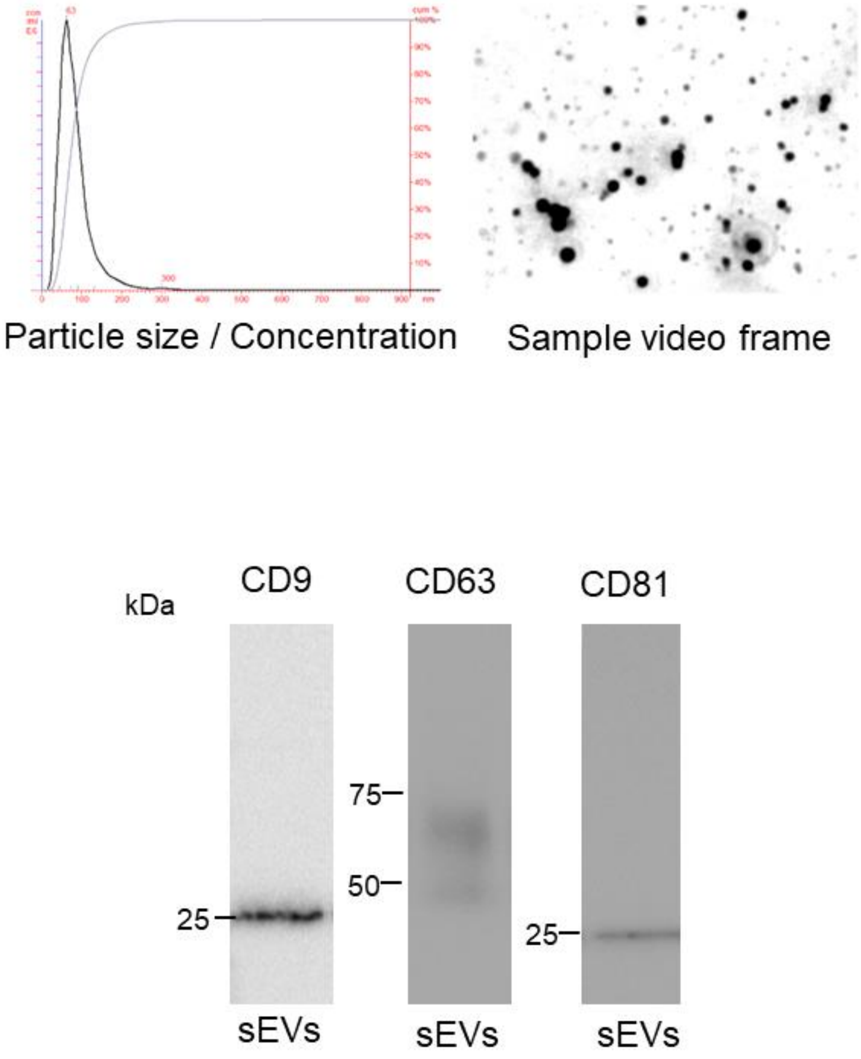

Supplementary Figure 1. Characterization of sEVs by (**A**) size and (**B**) surface markers CD9, CD63, and CD81 analyzed by western blotting.

Supplementary Figure 2

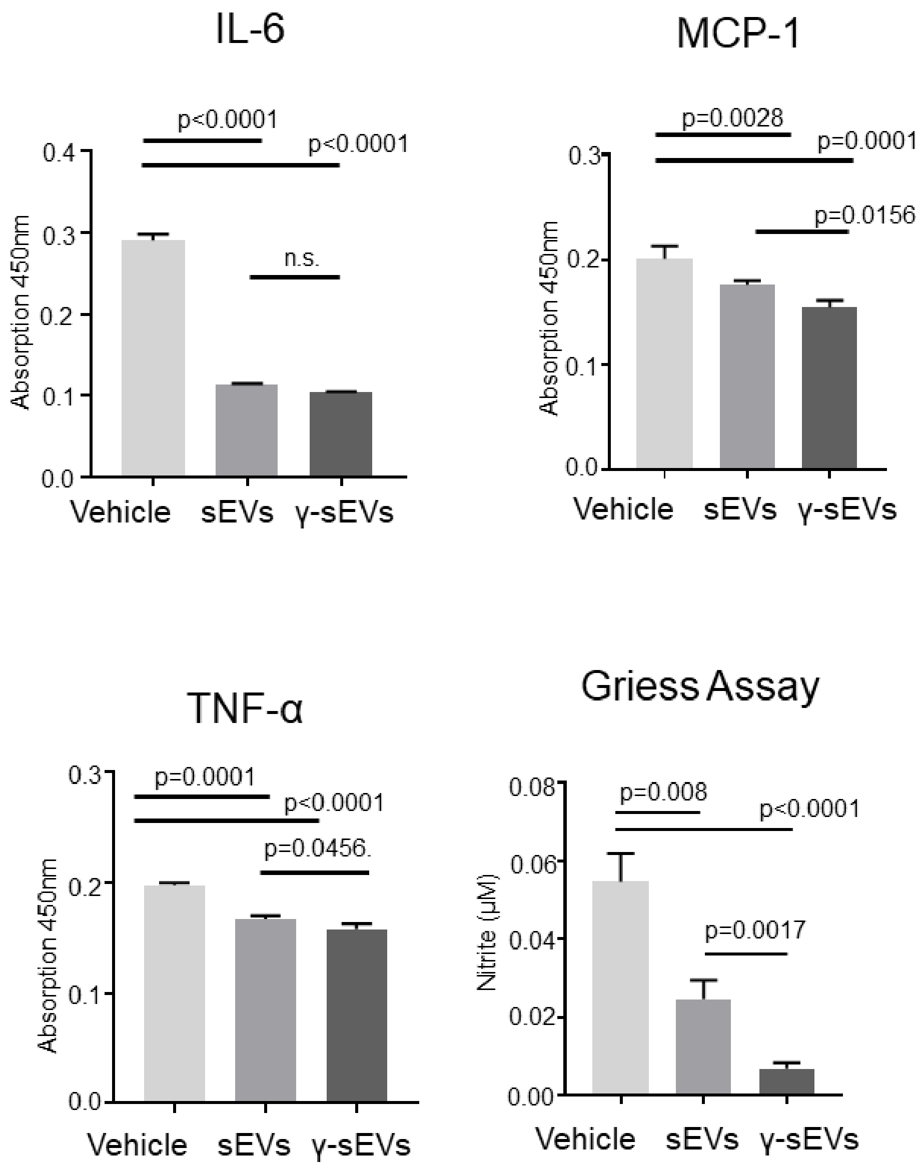

Supplementary Figure 2. ELISA and Griess assay of the macrophage culture supernatant after addition of vehicle, AD-MSC-sEVs (sEVs), and AD-MSC-γ-sEVs (γ-sEVs). Data are presented as means ± SD; *n* = 3 per experiment.

Supplementary Figure 3

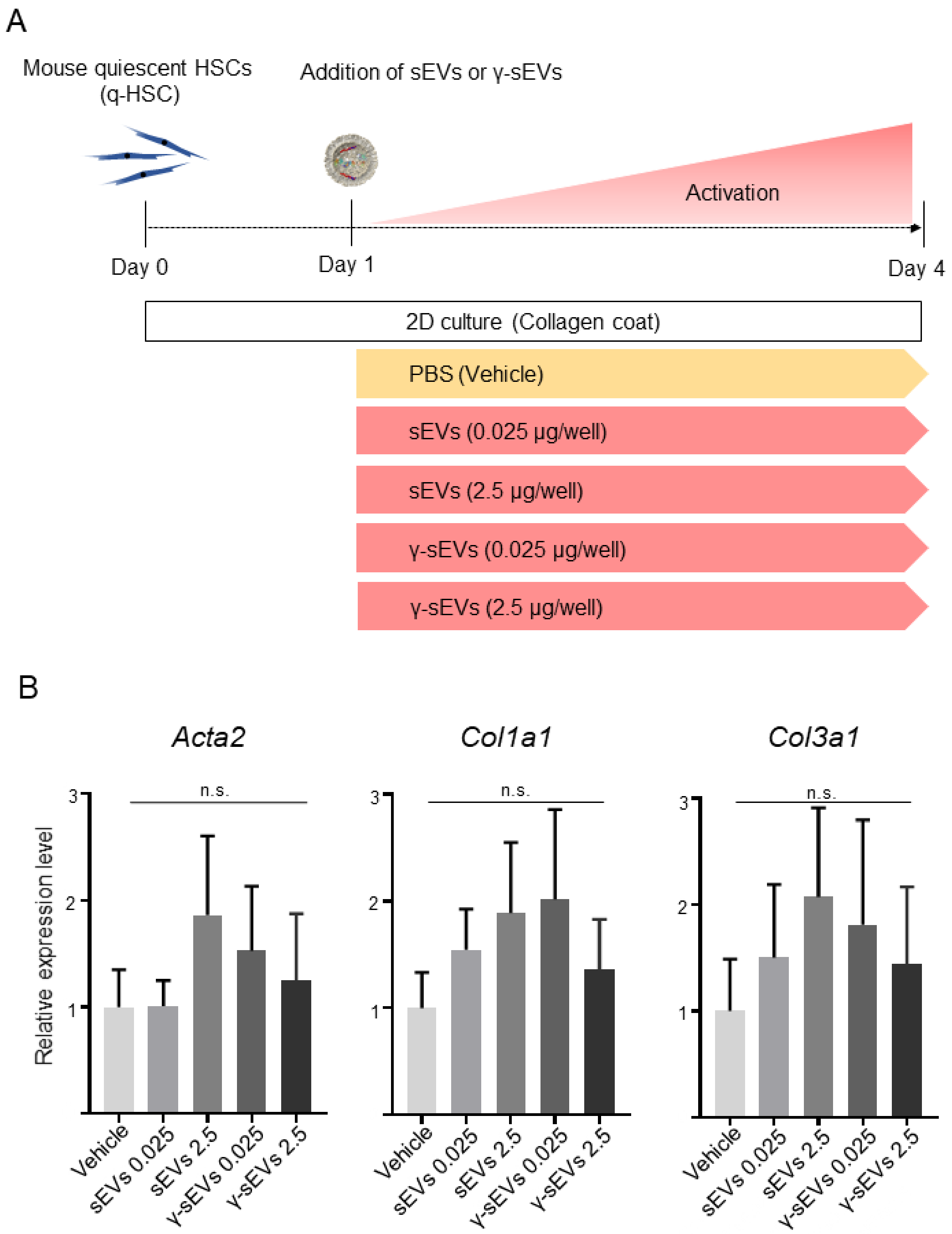

Supplementary Figure 3. Effects of AD-MSC-sEVs (sEVs) and AD-MSC-γ-sEVs (γ-sEVs) on HSC activation. (**A**) Schematic of experimental design. (**B**) Changes in the mRNA levels of *Acta2*, *Col1a1*, and *Col3a1*. Data are presented as means ± SD; $n = 3$ per experiment.

Supplementary Figure 4

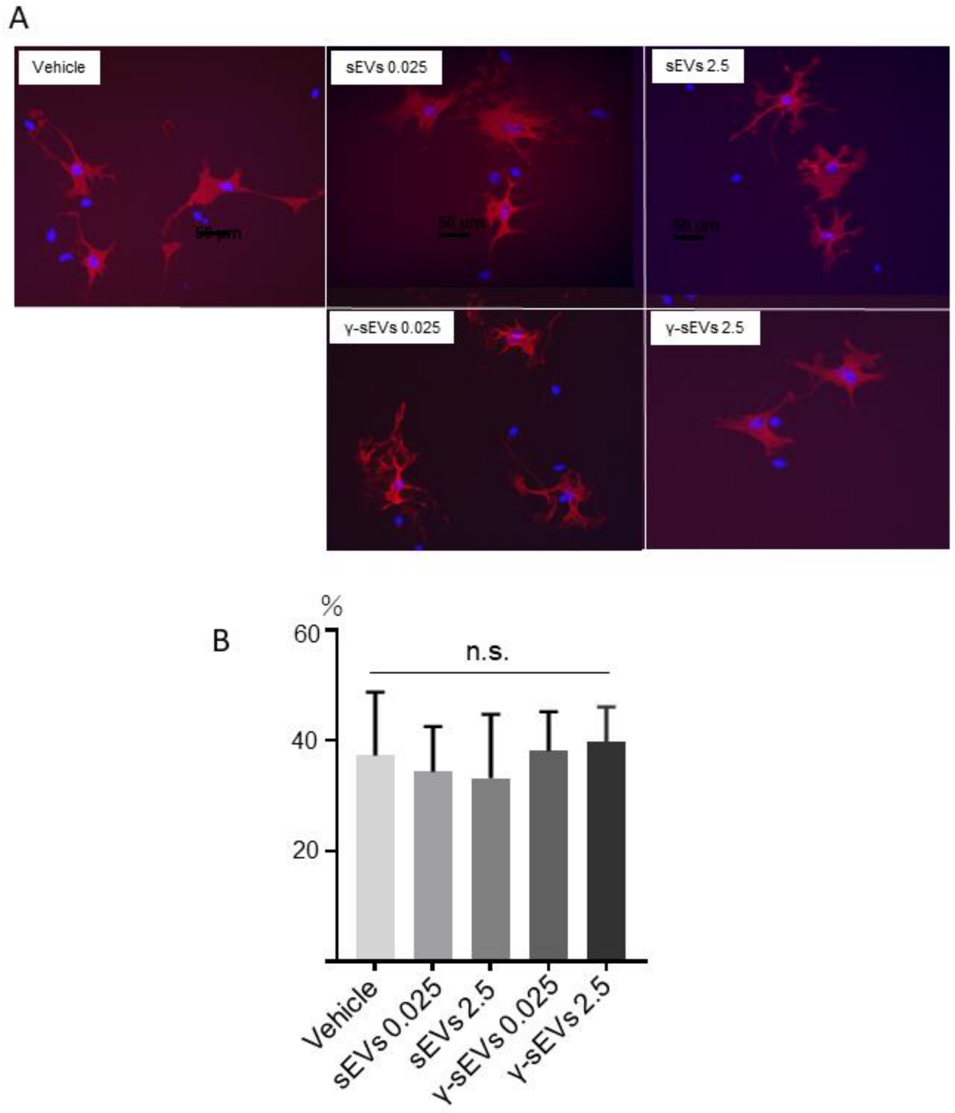

Supplementary Figure 4. α-SMA expression in cells cultured with vehicle, AD-MSC-sEVs (sEVs), and AD-MSC-γ-sEVs (γ-sEVs). (**A**) α-SMA positive cells (4 days after adding vehicle, sEVs and γ-sEVs) are stained red. (**B**) Frequency of α-SMA positive cells. Data are presented as means ± SD; *n* = 3 per experiment.

Supplementary Figure 5

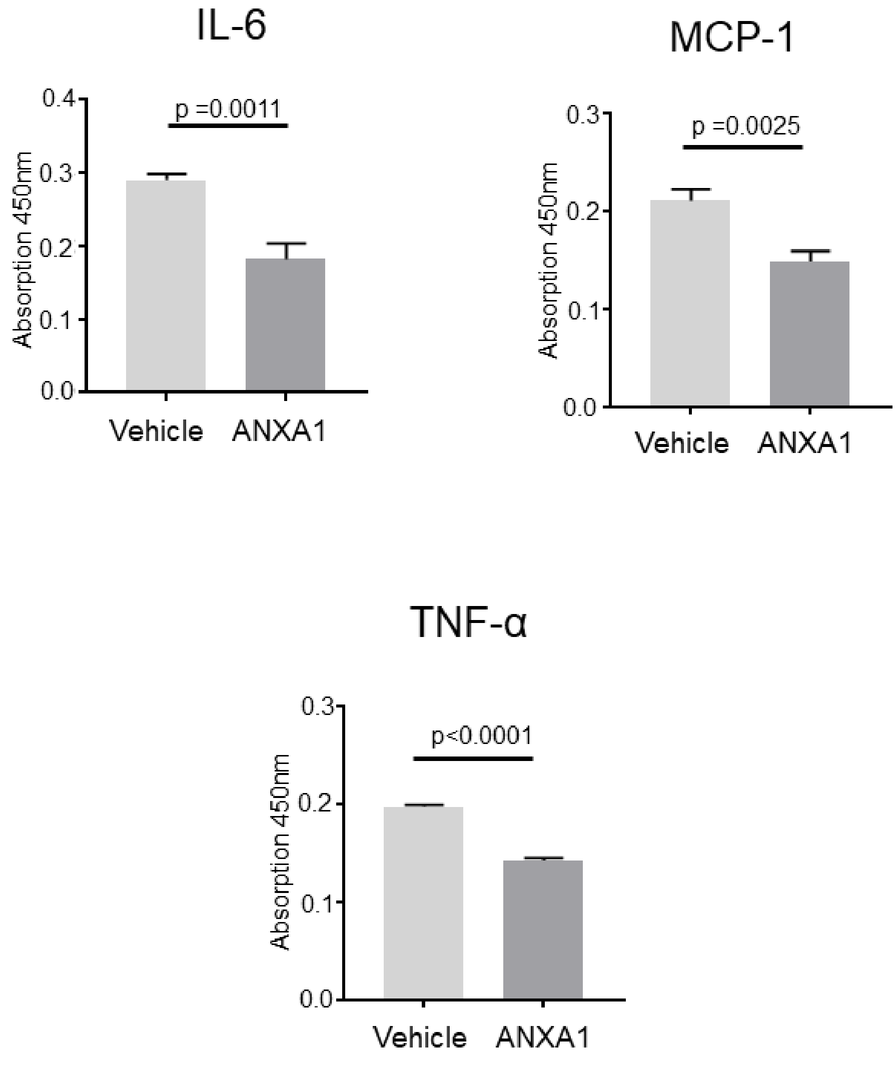

Supplementary Figure 5. ELISA of macrophage culture supernatant after addition of annexin A1. Data are presented as means ± SD; $n = 3$ per experiment.

Supplementary Figure 6

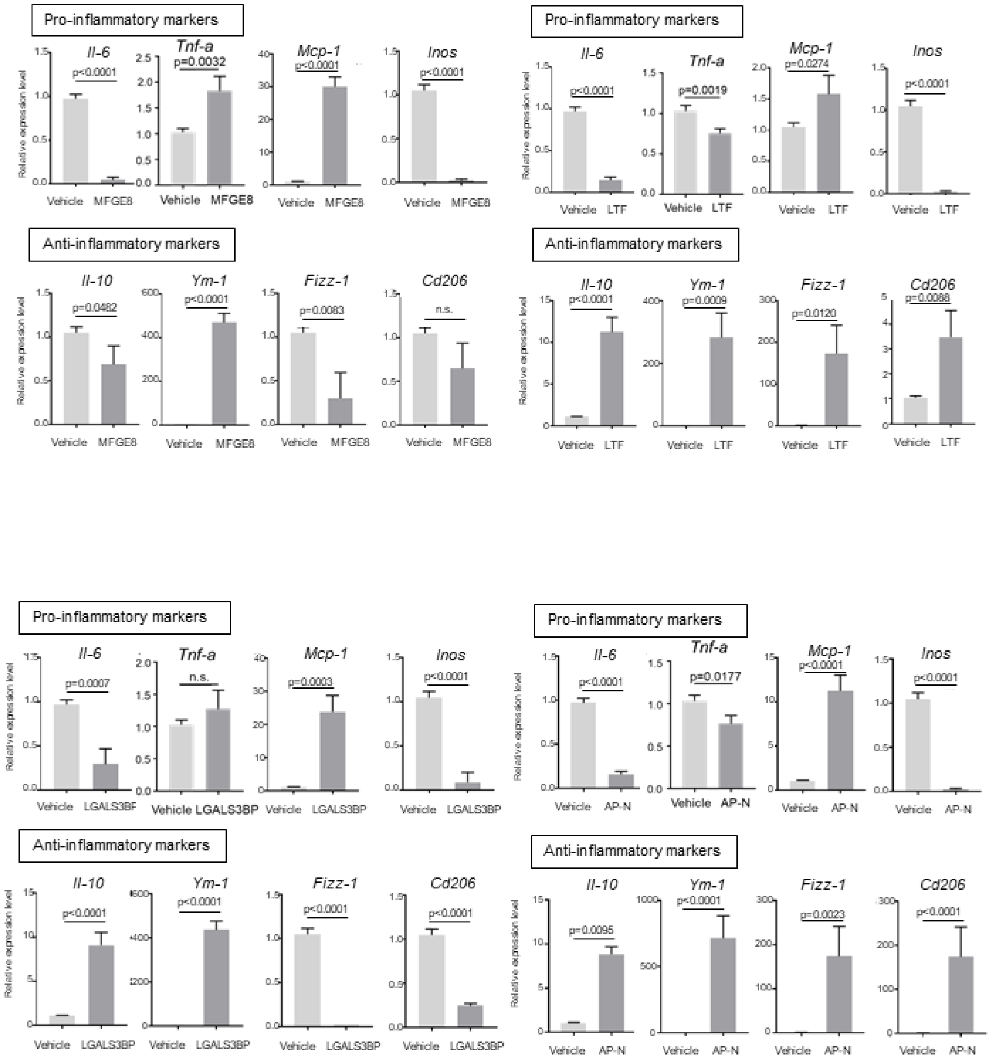

Supplementary Figure 6. Proteomic analysis of AD-MSC-sEVs (sEVs) or AD-MSC-γ-sEVs (γ-sEVs). The change in macrophage polarity after treatment with recombinant human lactadherin (MFGE8), recombinant human lactoferrin (lactotransferrin: LTF), recombinant galectin-3 binding protein (LGALS3BP), and recombinant human aminopeptidase N/CD13 (AP-N) is shown. Data are presented as means ± SD; *n* = 5 per experiment.

Supplementary Figure 7

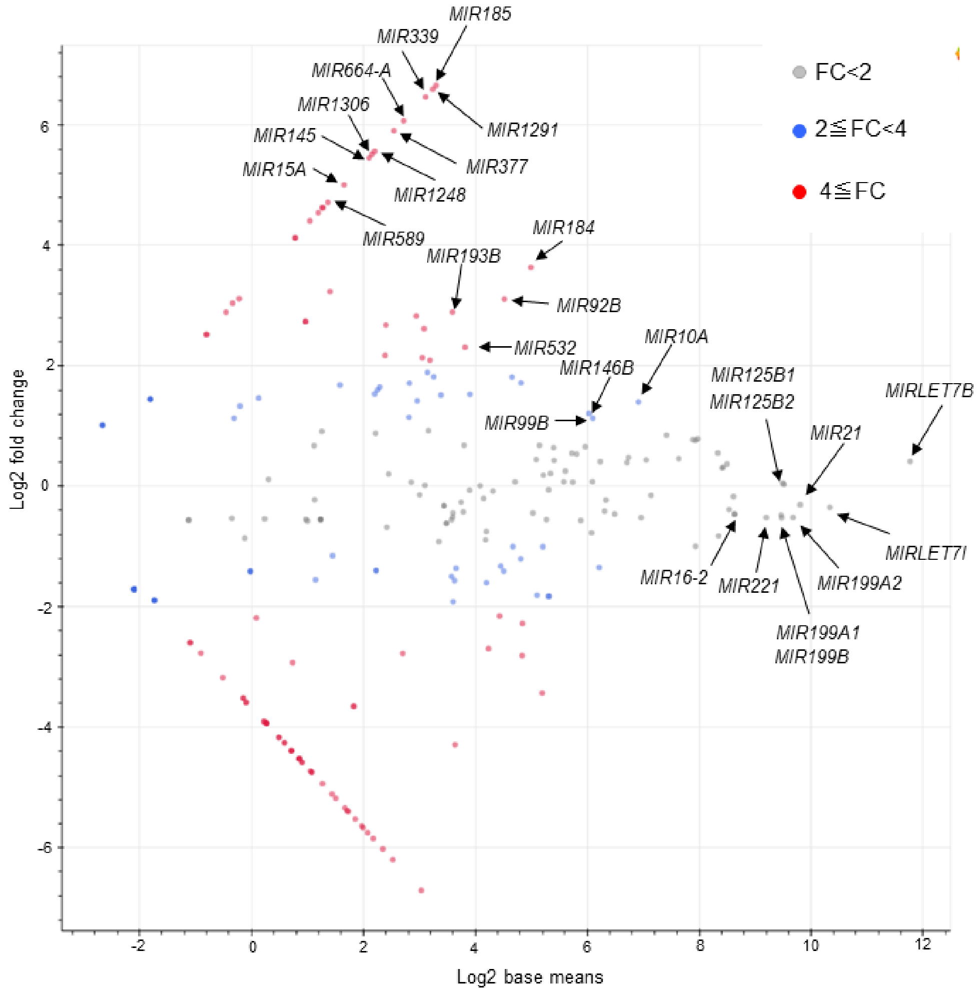

Supplementary Figure 7. Changes in miRNA levels in AD-MSC-γ-sEVs (γ-sEVs) compared to those in AD-MSC-sEVs (sEVs). Differences in the expression of miRNA between γ-sEVs and sEVs by MA-plot, in which data are shown as log2 transformed values. The X-axis shows the average quantitated value across the data and the Y axis shows the difference between γ-sEVs and sEVs. Silver, blue, and red dots represent miRNAs that changed log2FC < 2, 2 ≦ log2FC < 4, and 4 ≦ log2FC, respectively. FC; fold-change.

Supplementary Figure 8

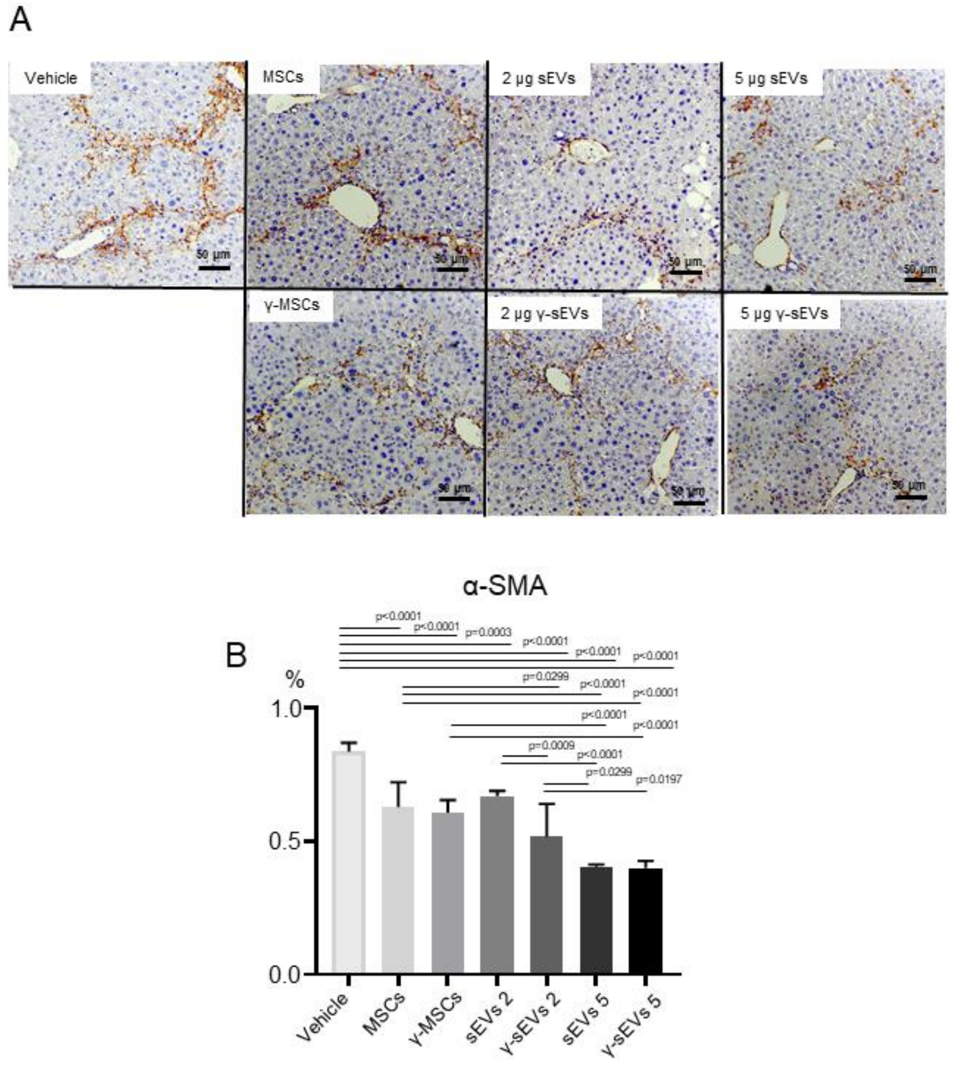

Supplementary Figure 8. α-SMA expression after injecting vehicle, AD-MSC, IFN-γ preconditioned AD-MSC (γ-AD-MSC), AD-MSC-sEVs (sEVs; 2 and 5μg), and AD-MSC-γ-sEVs (γ-sEVs; 2 and 5μg). (A) α-SMA positive cells are stained brown. (B) Frequency of α-SMA positive cells. Data are presented as means ± SD; *n* = 3 per experiment.

Supplementary Figure 9

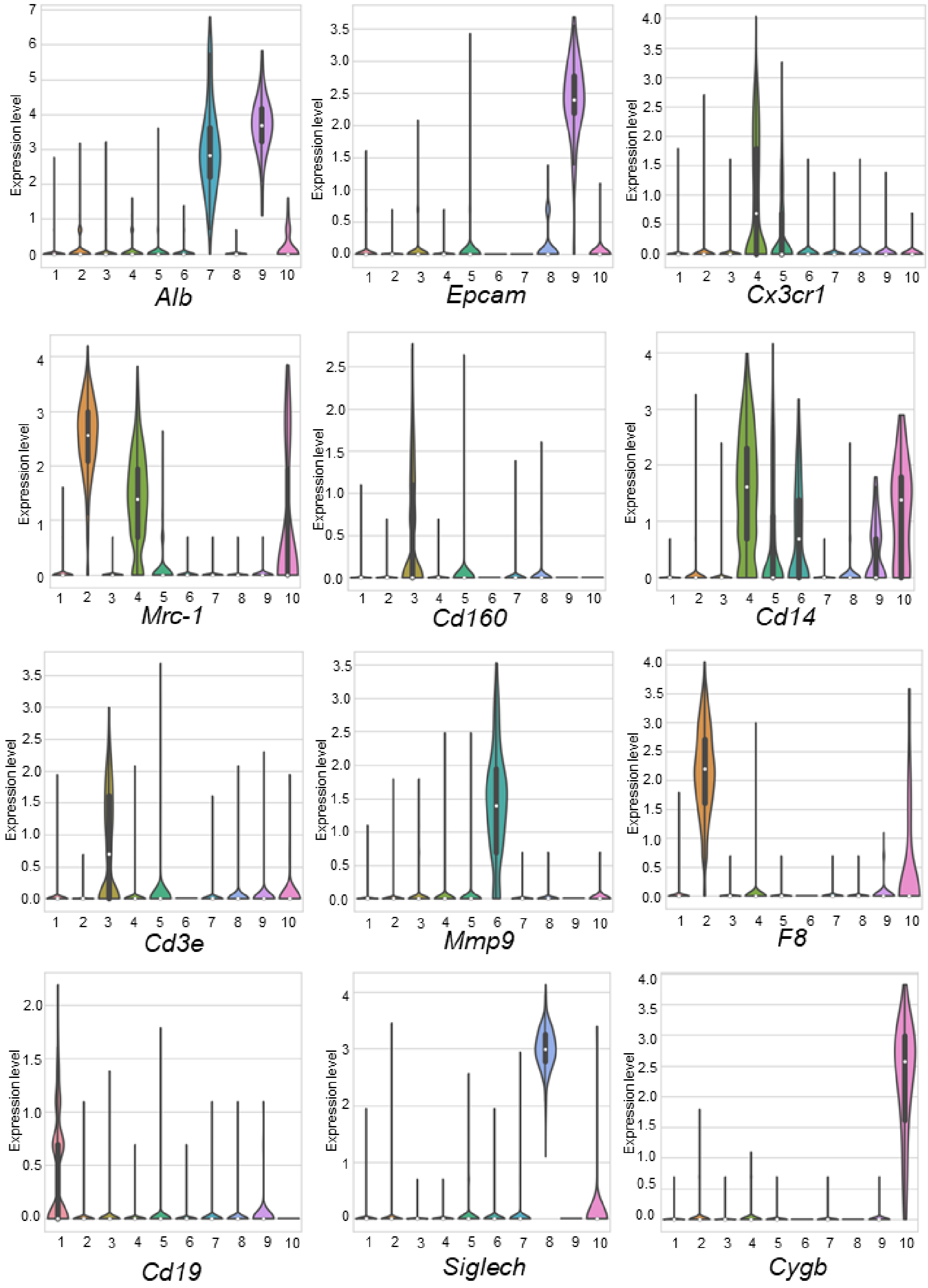

Supplementary Figure 9. Violin plots of data from single-cell transcriptome analysis. The distributions of *Alb, Epcam, Cx3cr1, Mrc-1, Cd160, Cd14, Cd3e, Mmp9, F8, Cd19, Siglech,* and *Cygb*-expressing cells are shown.

Supplementary Figure 10

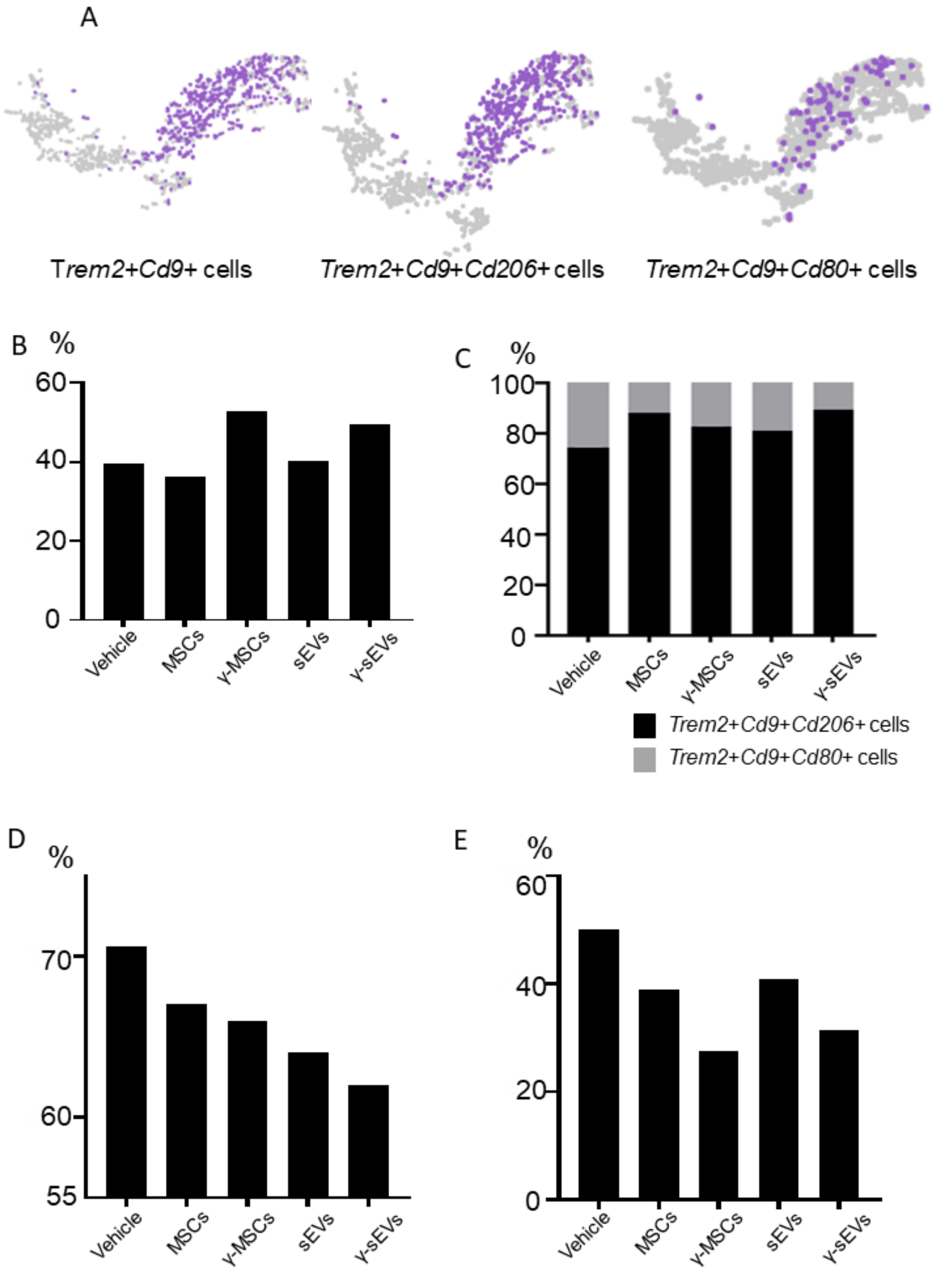

Supplementary Figure 10. Analysis of scar associated macrophages. (A) Distribution of *Trem2+Cd9+* cells, *Term2+Cd9+Cd206+* cells, and *Trem2+Cd9+Cd80+* cells. (B) Frequency of *Trem2+Cd9+* cells after injecting vehicle, AD-MSC (MSC), IFN-γ preconditioned AD-MSC (γ-MSC), AD-MSC-sEVs (sEVs), and AD-MSC-γ-sEVs (γ-sEVs). (C) Frequency of Cd80+ and Cd206+ cells in *Trem2+Cd9+* cells. (D) Frequency of *Il-1b+* cells and (E) frequency of *Tnf-a+* cells in *Trem2+Cd9+* cells.

Supplementary Figure 11

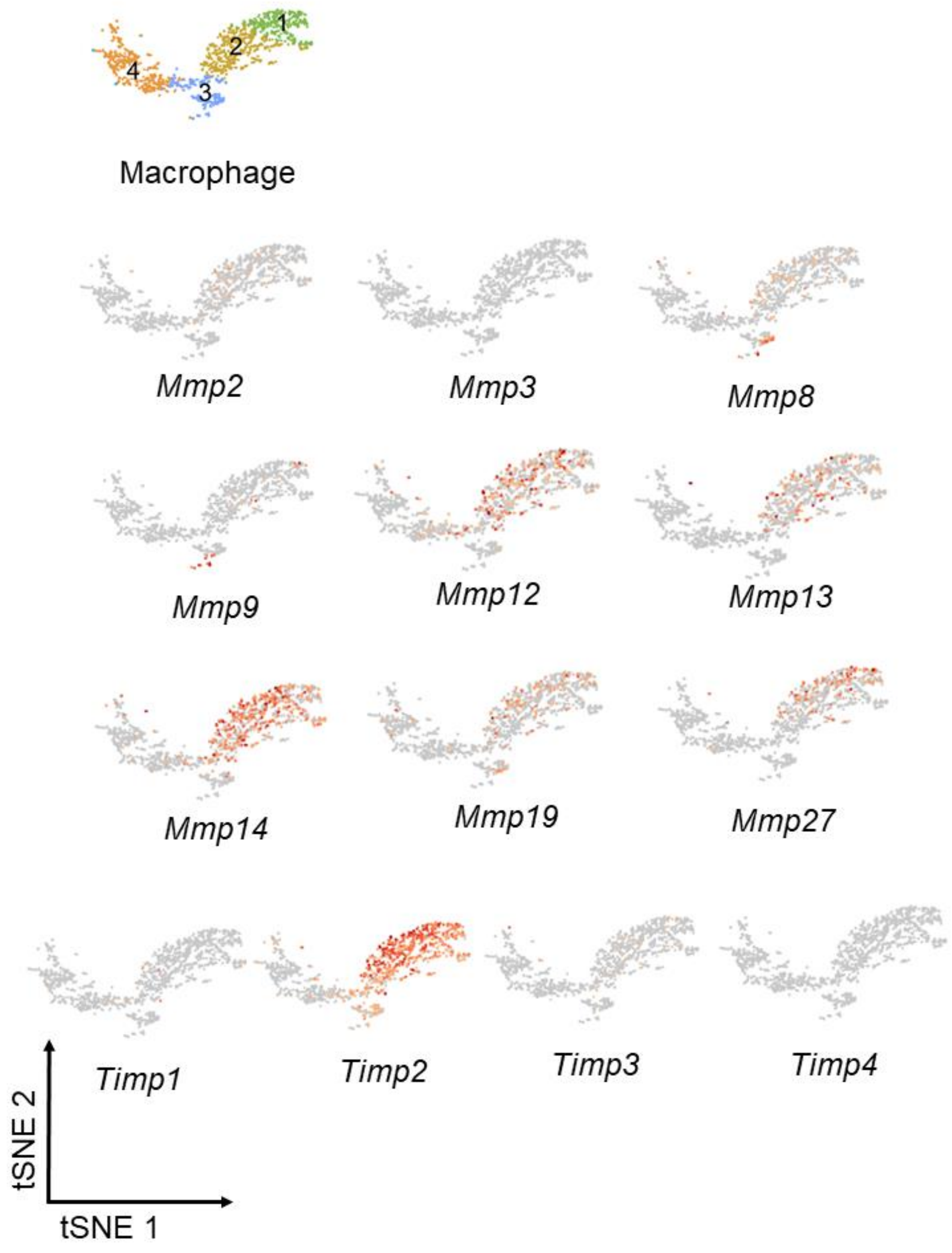

Supplementary Figure 11. *Mmp* and *Timp* expression in selected macrophage populations.

Un-cropped images

CD9

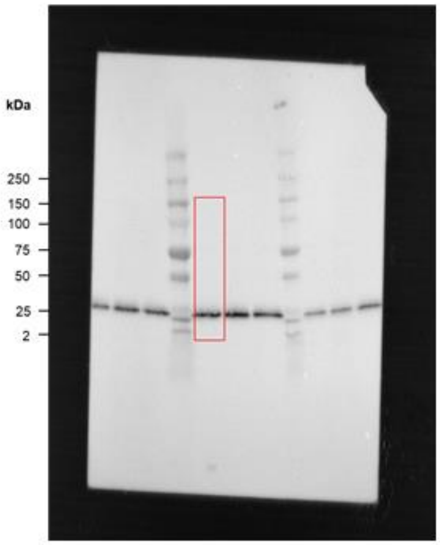

CD63

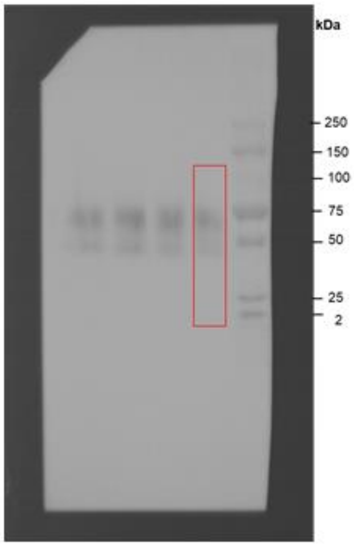

CD81

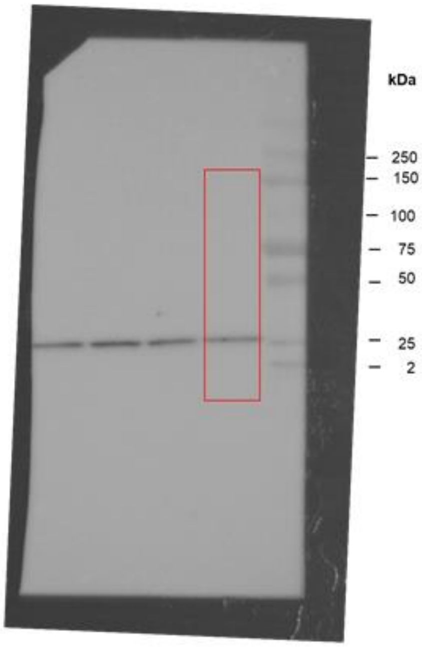

Supplementary Movie 1. Motility of macrophages after PBS (vehicle), AD-MSC-sEV (sEV), and AD-MSC-γ-sEV (γ-sEV) injections. Motility of GFP^+ macrophages were observed 240 min after addition of vehicle, sEVs, and γ-sEVs. Green, GFP-positive cells. Scale bar, 50 μm. Playback speed, 300×.

Supplementary Movie 2. Phagocytosis of macrophages after PBS (vehicle), AD-MSC-sEV (sEV), and AD-MSC-γ-sEV (γ-sEV) injection *in vitro*. sEVs, γ-sEVs, and pHrodo™ Green Zymosan Bioparticles™ conjugates were added 120 min after addition of vehicle, and phagocytic abilities of $DsRed^+$ macrophages were observed. Green, pHrodo™ Green Zymosan Bioparticles™ conjugates. Red, $DsRed^+$-positive cells. Scale bar, 50 μm. Playback speed, 600×.

Supplementary Movie 3. Intravital liver imaging of CCl_4-damaged CX_3CR1-EGFP mice treated with PBS (vehicle), AD-MSC-sEVs (sEVs), or AD-MSC-γ-sEVs (γ-sEVs) *in vivo*. CCl4 was injected intraperitoneally twice per week for 8 weeks. Intravital imaging was performed 24 hours after intravenous injection of vehicle, sEVs, and γ-sEVs. Sequential images of the same visual field are shown. Green, CX_3CR1-EGFP-positive cells. Red, blood vessels visualized by intravenous injection of Texas Red-conjugated 70 kDa dextran. Scale bar, 50 μm. Playback speed, 300×.
